# Huddling behavior regulate adaptive thermogenesis in Brandt’s voles (*Lasiopodomys brandtii*)

**DOI:** 10.1186/s13578-025-01391-0

**Published:** 2025-04-23

**Authors:** Min Liu, De-Sheng Zou, Xue-Ying Zhang, De-Hua Wang

**Affiliations:** 1 School of Life Sciences, Shandong University, Qingdao, 266237 China; 2 State Key Laboratory of Integrated Management of Pest Insects and Rodents, Institute of Zoology, Chinese Academy of Sciences, Beijing, 100101 China

**Keywords:** Huddling, Cold acclimation, Hypothalamus, Brown adipose tissue, Thermogenesis, Brandt’s voles (*Lasiopodomys brandtii*)

## Abstract

**Background:**

Brown adipose tissue (BAT) is the main site of non-shivering thermogenesis (NST) in small mammals, playing an important role in maintaining body temperature and energy balance. Huddling is a behavioral strategy for small rodents to save energy and improve the survival under cold environments. However, the way of huddling behavior influence on hypothalamus, which regulate BAT thermogenesis in small mammals is rarely illustrated. We used male Brandt’s voles (*Lasiopodomys brandtii*) to explore the possible regulation mechanisms in BAT thermogenesis by the way of cold acclimation and huddling behavior.

**Results:**

There is a strong relationship between huddling behavior and NST in BAT. The hypothalamus, which is impacted by huddling behavior, influences PPAR signaling pathway in the BAT, and induces thermogenesis through Calcium signaling pathway. PPAR pathway causes crosstalk among NF-κB signaling pathway, Thermogenesis and Fatty acid metabolism to perform functions for thermogenesis.

**Conclusions:**

The results suggest that huddling behavior can modulate adaptive thermogenesis in BAT. Cold acclimation and huddling had a synergistic effect on the regulation of thermogenic function, the hypothalamus mediates thermogenic changes in BAT induced by huddling behavior. In BAT, the specific pathway of thermogenesis is as follows: TRAF6-PPARγ-UCP1-SUCLG1.

**Supplementary Information:**

The online version contains supplementary material available at 10.1186/s13578-025-01391-0.

## Background

Brown adipose tissue (BAT) is a major organ for non-shivering thermogenesis (NST) in small mammals and infants [1], by consuming glucose and provide a heat source for animals to protect them from cold stress. In the past few decades, many studies have identified positive or negative regulatory factors involved in the development of brown adipocytes [2]. The brain integrates ambient temperature signals and regulates brain regions for thermogenesis, ultimately providing coordinated output responses [3–5]. Among the numerous brain regions involved in this function, the hypothalamus plays a crucial role. The hypothalamus controls lipid and glucose metabolism in peripheral tissues through the autonomous nervous system (ANS), including BAT thermogenesis and WAT browning [3–7], and the increase in thermogenesis is attributed to the increased expression of uncoupling protein 1 (UCP1) [8]. Peroxisome proliferator-activated receptors (PPARs) have emerged as integrators of inflammatory and metabolic signalling networks [9]. PGC1α (PPARγ coactivator 1α) is described as a coactivator of PPARγ. Together, these two factors regulate the expression of UCP1 and promote thermogenesis in BAT [10].

Huddling may be particularly important in conserving energy for species that are subject to low ambient temperatures, for social species, and species with poorly insulated, or have high surface-to-volume ratios. Especially for wild animals, huddling has the benefits for the survival rate of members and energy consumption at low ambient temperatures [11–13]. Huddling is not only a physical process, it also plays an important role in physiology. This mechanism can explain energy saving relies on adjustment in body temperature [14]. For most mammals and birds, huddling individuals maintain a higher body temperature than isolated counterparts, suggesting that huddling may act as a warming mechanism, potentially due to reduction in heat loss [15, 16]. Nevertheless, some species lower their body temperatures when they are huddling, which enables them to save energy by reducing metabolic heat production. Great snow geese goslings (*Chen caerulescens atlantica*) reduced body temperature by 0.8°C during huddling [17]. When gray mouse lemurs (*Microcebus murinus*) exposed to cold temperatures and a calorie-restricted diet, compared with isolated males, two males in a group increased duration of torpor (daily torpor is as an energy-saving strategy, they rest in small groups in their nest), but torpor depth was similar [18, 19]. These examples suggest that huddling plays a role in thermoregulation as a physiological process.

Previous studies in our laboratory have found that huddling behavior can reduce resting metabolic rates (RMR) and non-shivering thermogenesis (NST) in Brandt’s voles (*Lasiopodomys brandtii*) [20]. Cold temperature decreased cell proliferation in the hypothalamus, while huddling can mitigate this effect. That is, in cold environment, the number of new born cells in functional regions of hypothalamus in huddling voles is higher than solitary one (unpublished data from research group). These results suggest that huddling behavior plays a significant role in the functioning of BAT and hypothalamus. Brandt’s vole is a nonhibernating rodent species, which are widely distributed in steppe zone of Mongolia, south east of Baikal region in Russia and Inner Mongolian grasslands in the north of China [21]. The characteristics of its habitat are extremely cold and dry in winter, and the soil is deeply frozen. Brandt’s voles show seasonal variations in energy metabolism and thermoregulation in response to seasonal environments [8, 22], which include the increase of BAT mass and NST in winter [23, 24]. The relationship between huddling behavior and BAT thermogenesis has been reported in studies. When mice were exposed to cold temperatures, those living in groups exhibited reduced BAT development compared to solitary mice [25]. The previous results of our research group also proved that huddling has the ability to influence the energy metabolism of Brandt’s voles. However, it is still unclear how huddling behavior affects BAT in a thermogenic role. In addition, although it has been clarified that the hypothalamus can regulate BAT thermogenesis in mammals, it is still unclear how the hypothalamus interacts with the BAT under the influence of cold and huddling behavior and through what pathways to exert thermogenic function, ultimately achieving the goal of reducing energy metabolism. In this study, we expand on our previous research to investigate the mechanisms of behavioral regulation of thermogenesis. Explain how behavior and temperature affect the hypothalamus and BAT to play a regulatory and thermogenic role. The purpose of this study is to reveal the adaptation of animals to cold at multiple levels, and ultimately illustrate the regulatory mechanisms of animal cold temperature adaptation.

## Results

We set up 4 treatments: cold (at 4 ± 1°C) and huddling (CH), cold and separated (CS), warm (at 23 ± 1°C) and huddling (WH), warm and separated (WS). Huddling means the voles cluster together except during free time. Phenotype, physiological and molecular indicators were quantified and the results combined with transcriptomics data, to express the influence of huddling behavior on the control of BAT thermogenesis.

### Effects of cold and huddling on metabolic phenotype

**Fig. 1:**
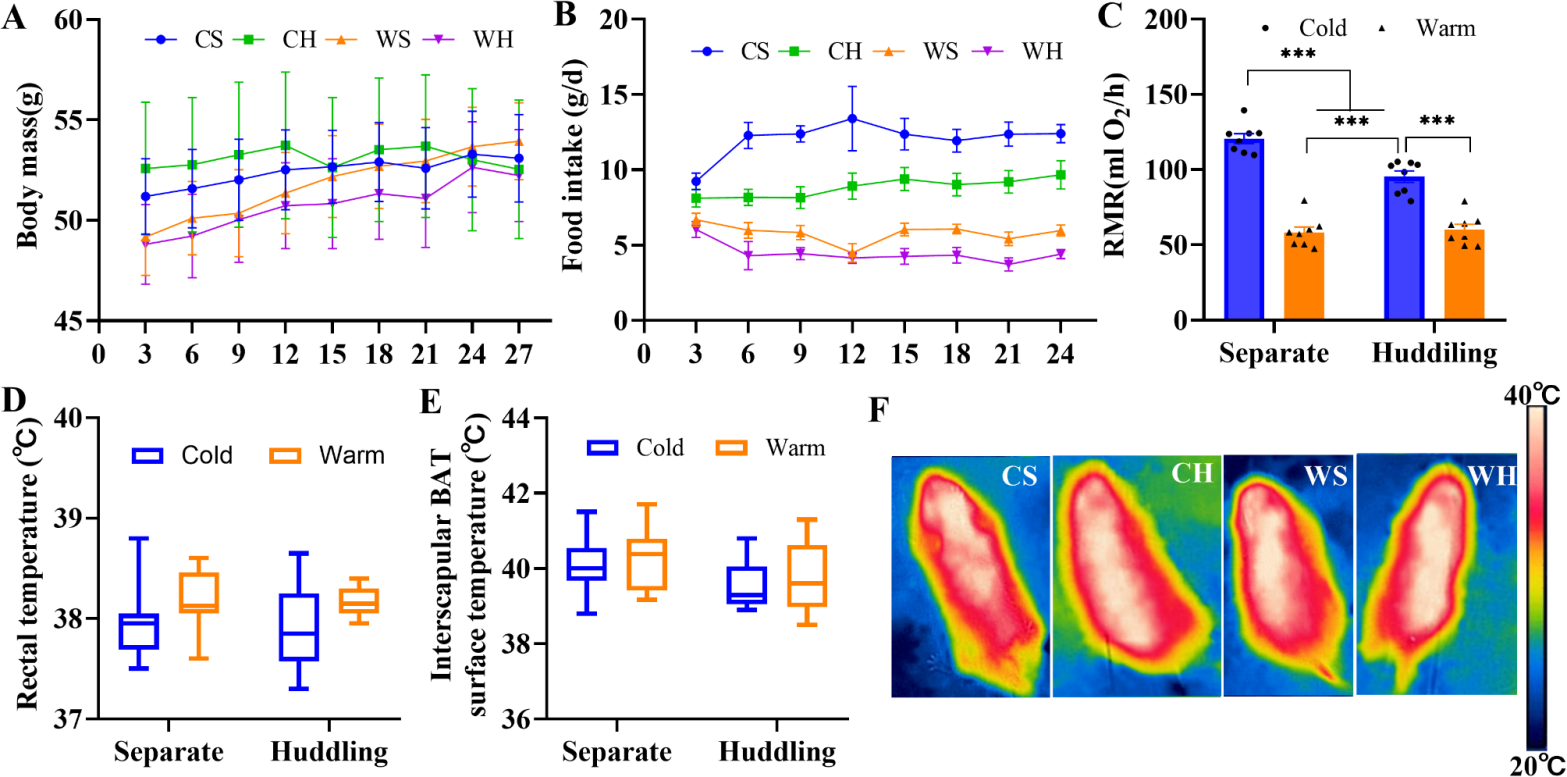
Basic metabolic phenotype of Brandt’s voles affected by temperature and huddling. Body mass (**A**), food intake (**B**), resting metabolic rates (RMR, **C**), rectal temperature (**D**), surface temperature of interscapular BAT (**E**) and infrared pictures (**F**). Values are means ± SEM. *, *p* < 0.05, ***, *p* < 0.001. The X-axis labels in Fig. (**A**) and (**B**) represent days

During the acclimation of the voles, there was no significant difference in body mass among the groups (F_3,74_=0.452, *p* = 0.716, Fig. 1A), but grouping led to a significant impact on food intake, cold significantly increase food intake (F_3,16_=37.466, *p*<0.001, Fig. 1B). Both temperature (F_1,28_=186.929, *p*<0.001) and huddling (F_1,28_=10.732, *p* = 0.003) affected RMR, and the two factors had an interaction (F_1,28_=14.419, *p* = 0.001, Fig. 1C). Neither ambient temperature nor huddling had a significant effect on the rectal temperature (Temperature: F_1,28_=3.995, *p* = 0.055; Huddling: F_1,28_=0.114, *p* = 0.738, Fig. 1D) or interscapular BAT (iBAT) surface temperature (Temperature: F_1,28_=0.406, *p* = 0.529; Huddling: F_1,28_=3.428, *p* = 0.075, Fig. 1E and F) of the voles.

However, the current results only demonstrate huddling behavior and cold environment have an impact on non-shivering thermogenesis, but do not provide insight into how huddling regulates BAT. Therefore, we performed transcriptome analyses based on phenotypic data to obtain further results.

### Analysis of differentially expressed genes

**Fig. 2:**
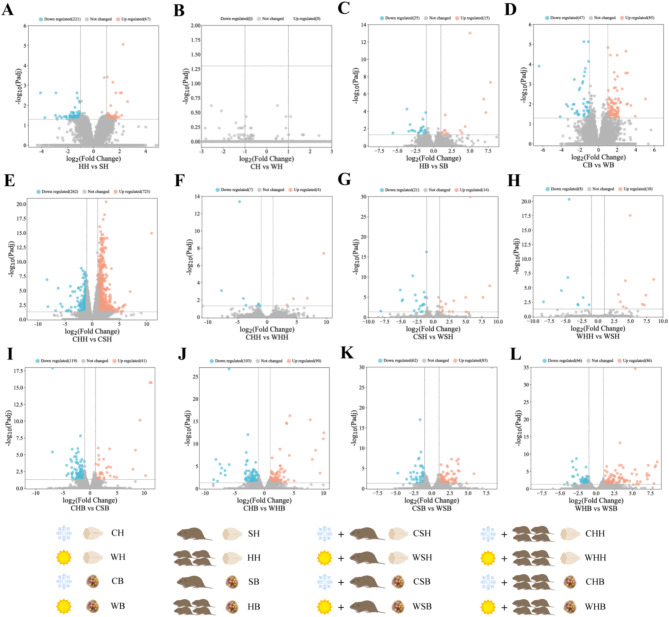
Volcano map of differentially expressed genes (DEGs) in the hypothalamus and brown adipose tissue of Brandt’s vole under different environmental conditions. Volcanic maps of DEGs in different ways: huddling hypothalamus versus separate hypothalamus (**A**), cold hypothalamus versus warm hypothalamus (**B**), huddling BAT versus separate BAT (**C**), cold BAT versus warm BAT (**D**), cold huddling hypothalamus versus cold separate hypothalamus (**E**), cold huddling hypothalamus versus warm huddling hypothalamus (**F**), cold separate hypothalamus versus warm separate hypothalamus (**G**), warm huddling hypothalamus versus warm separate hypothalamus (**H**), cold huddling BAT versus cold separate BAT (**I**), cold huddling BAT versus warm huddling BAT (**J**), cold separate BAT versus warm separate BAT (**K**), warm huddling BAT versus warm separate BAT (**L**). Red represents up-regulated DEGs, green represents down-regulated DEGs, and gray represents no significance

We identified DEGs in the groups based on the comparison among different groups. Volcanic maps were used to determine up or down-regulation of gene expression. The negative logarithm log10 (*p*-adjust value) is converted as the vertical axis. Firstly, we determined the effects of a single factor such as temperature or huddling. In hypothalamus, huddling causes up-regulation of 67 genes and down-regulation of 221 genes (Fig. 2A). However, temperature does not lead to up or down-regulation of gene expression (Fig. 2B). In BAT, huddling results in 15 up-regulated genes and 25 down-regulated genes (Fig. 2C). Cold results in 85 up-regulated genes and 47 down-regulated genes (Fig. 2D). Then, we focused on the simultaneous effects of cold and huddling on DEGs. Specifically, the up or down-regulated DEGs are obtained by controlling the change of a factor and comparing between groups.

For the hypothalamus, under cold conditions, huddling resulted in 723 DEGs being up-regulated and 262 DEGs being down-regulated compared to the separated group (Fig. 2E). When voles huddled, compared to warm conditions, the cold can cause 4 DEGs to be up-regulated and 7 DEGs to be down-regulated (Fig. 2F). When voles were separated, compared to warm temperatures, the cold caused 14 DEGs to be upregulated and 21 DEGs downregulated (Fig. 2G). Under warm conditions, compared to the separated group, huddling resulted in an upregulation of 10 DEGs and a downregulation of 8 DEGs (Fig. 2H).

For BAT, under cold conditions, huddling resulted in 41 DEGs being upregulated and 119 DEGs being downregulated compared to the separated group (Fig. 2I). When animals huddled, compared to warm conditions, the cold caused 90 DEGs to be upregulated and 103 DEGs to be downregulated in voles (Fig. 2J). When animals were separated, compared to warm temperatures, the cold caused 85 DEGs to be upregulated and 62 DEGs to be downregulated (Fig. 2K). Under warm conditions, compared to separated group, huddling resulted in an upregulation of 86 DEGs and a downregulation of 66 DEGs (Fig. 2L). The number of DEGs is closely related to function. Whether it is a single factor (temperature or huddling) or a combination of two factors (control for single factor changes), with regard to results of DEGs, the impact of huddling on the hypothalamus is more significant than the temperature effect. Nevertheless, for BAT, the temperature effect is more pronounced. Therefore, based on the number of DEGs, it is hypothesised that the effect of huddling is primarily on hypothalamic function, while temperature exerts a major effect on BAT function.

### Enrichment analysis

#### Enrichment analysis of KEGG pathway of DEGs in hypothalamus

**Fig. 3:**
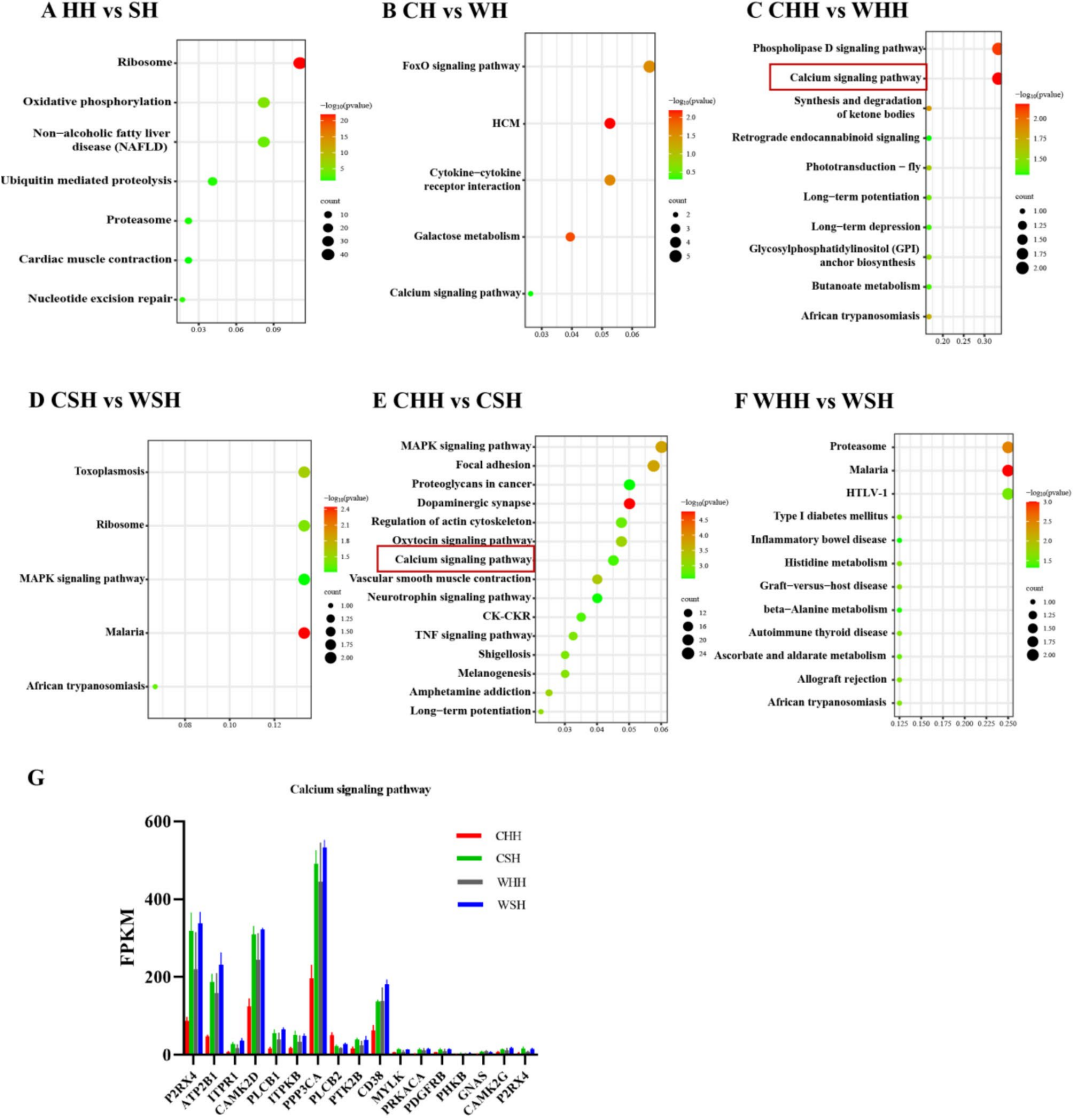
KEGG pathway map of differentially expressed genes (DEGs) in hypothalamus. KEGG pathways of DEGs by Huddling versus separate (**A**), Cold versus warm (**B**), Cold huddling versus warm huddling (**C**), Cold separate versus warm separate (**D**), Cold huddling versus cold separate (**E**), Warm huddling versus warm separate (**F**), FPKM of DEGs in Calcium signaling pathway under the influence of two factors (**G**). In the bubble chart, the size of the dot corresponds to the number of DEGs in the pathway. The larger the dot, the higher the count. The colour of the dot ranges from green to red, representing increasing significance of enrichment. The degree of enrichment is indicated by “-log10 (P value)”

We first analyzed the enrichment of DEGs under the influence of a single factor (temperature or huddling). The results indicate that due to the influence of huddling, DEGs mainly enriched in Ribosome and Oxidative phosphorylation pathways (Fig. 3A). Affected by temperature effect, DEGs mainly enriched in FoxO signaling pathway (Fig. 3B). On this basis, we continued to analyze the enrichment of DEGs under the simultaneous influence of temperature and huddling factors. The results showed that when voles huddled, DEGs mainly enriched in the Calcium signaling pathway under the influence of temperature (Fig. 3C). When voles were separated, DEGs mainly enriched in Ribosome under the influence of temperature (Fig. 3D). During low-temperature acclimation, DEGs enriched in various functional pathways such as Dopaminergic synapse, MAPK signaling pathway, TNF signaling pathway, Calcium signaling pathway, NF kappa B signaling pathway, and PI3K Akt signaling pathway under the influence of huddling (Fig. 3E). Under the influence of huddling, DEGs enriched in amino acid metabolism, such as Histidine and beta-Alanine metabolism, when animals were at room temperature (Fig. 3F). Regarding functional pathways, the Calcium signaling and Ribosome are the two KEGG enrichment pathways that repeat in different comparative ways. Ribosomes are factories for protein translation and energy consumption in cells. Except for mature red blood cells, all mammalian cells have ribosomes. Due to its lack of specificity, subsequent research mainly focuses on the Calcium signaling pathway. Next, statistical analysis was conducted on the expression abundance of DEGs in Calcium signaling pathway (Fig. 3G) to serve as the basis for subsequent screening of critical genes, DEGs in this pathway include CAMK2D (Calcium/calmodulin-dependent protein kinase II delta), ATP2B1 (Plasma membrane calcium transporting ATPase 1), CD38 (Cyclic ADP-ribose hydrolase 1) etc.

#### Enrichment analysis of KEGG pathway of DEGs in BAT

**Fig. 4:**
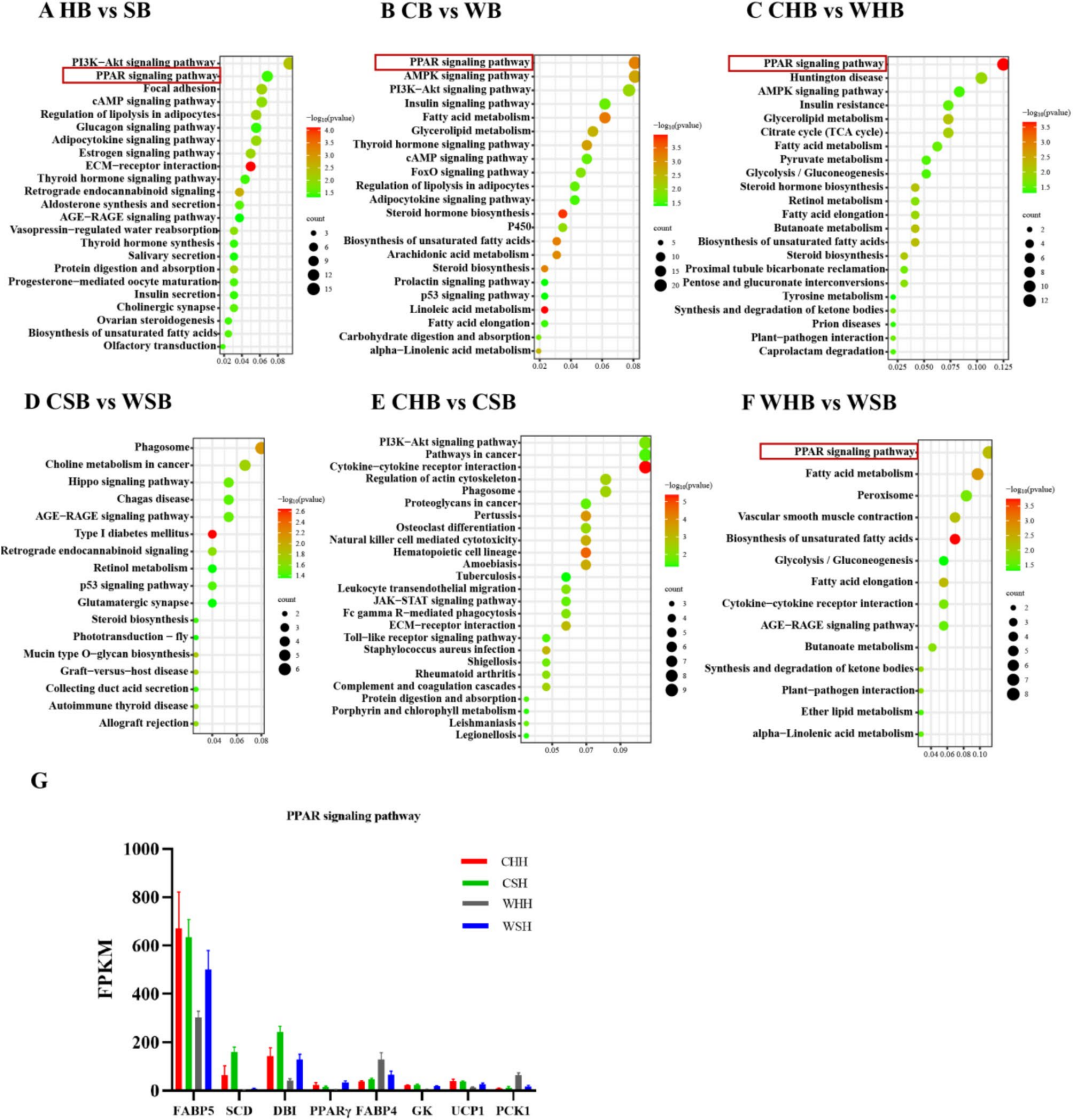
KEGG pathway map of differentially expressed genes (DEGs) in BAT. KEGG pathways of DEGs in BAT by Huddling versus separate (**A**), Cold versus warm (**B**), Cold huddling versus warm huddling (**C**), Cold separate versus warm separate (**D**), Cold huddling versus cold separate (**E**), Warm huddling versus warm separate (**F**), FPKM of DEGs in PPAR signaling pathway under the influence of two factors (**G**). In the bubble chart, the size of the dot corresponds to the number of DEGs in the pathway. The larger the dot, the higher the count. The colour of the dot ranges from green to red, representing increasing significance of enrichment. The degree of enrichment is indicated by “-log10 (P value)”

The KEGG pathway analysis for BAT corresponds to the hypothalamus. The first step is to assess the enrichment of DEGs under a single factor (either temperature or huddling). Due to huddling effect, DEGs are primarily concentrated in the PPAR signaling pathway, PI3K-Akt signaling pathway, cAMP signaling pathway, and Thyroid hormone signaling pathway (Fig. 4A). Influenced by ambient temperature, DEGs exhibit significant enrichment within the PPAR signaling pathway, Fatty acid metabolism pathway, AMPK signaling pathway, and Insulin signaling pathway (Fig. 4B). Similarly, we examined the enrichment of DEGs that were affected by temperature and huddling. The impact of temperature is that, when Brandt’s voles huddled, DEGs enriched in PPAR signaling pathway, Citrate cycle, Tyrosine metabolism, and Fatty acid metabolism (Fig. 4C), and when animals were separated, DEGs enriched in Glutamate synapses and Hippo signaling pathways(Fig. 4D). The effect of huddling is that, under cold temperature, DEGs enriched in PI3K-Akt signaling pathway, JAK-STAT signaling pathway, Toll-like receptor signaling pathway (Fig. 4E), and under warm temperature, DEGs enriched in PPAR signaling pathway, Fatty acid metabolism, Glycolysis/Gluconeogenesis (Fig. 4F). The pathways mentioned above are the KEGG enrichment pathways that are related to our research. Compared to the hypothalamus, DEGs in the BAT show a higher enrichment in functional pathways. The PPAR signaling pathway repeatedly appears in various comparisons, demonstrating its crucial function in BAT. Next, we examined the expression abundance of DEGs within the PPAR signaling pathway (Fig. 4G) to establish a foundation for identifying pivotal genes in subsequent screening.

### Weighted gene coexpression network analysis (WGCNA)

**Fig. 5:**
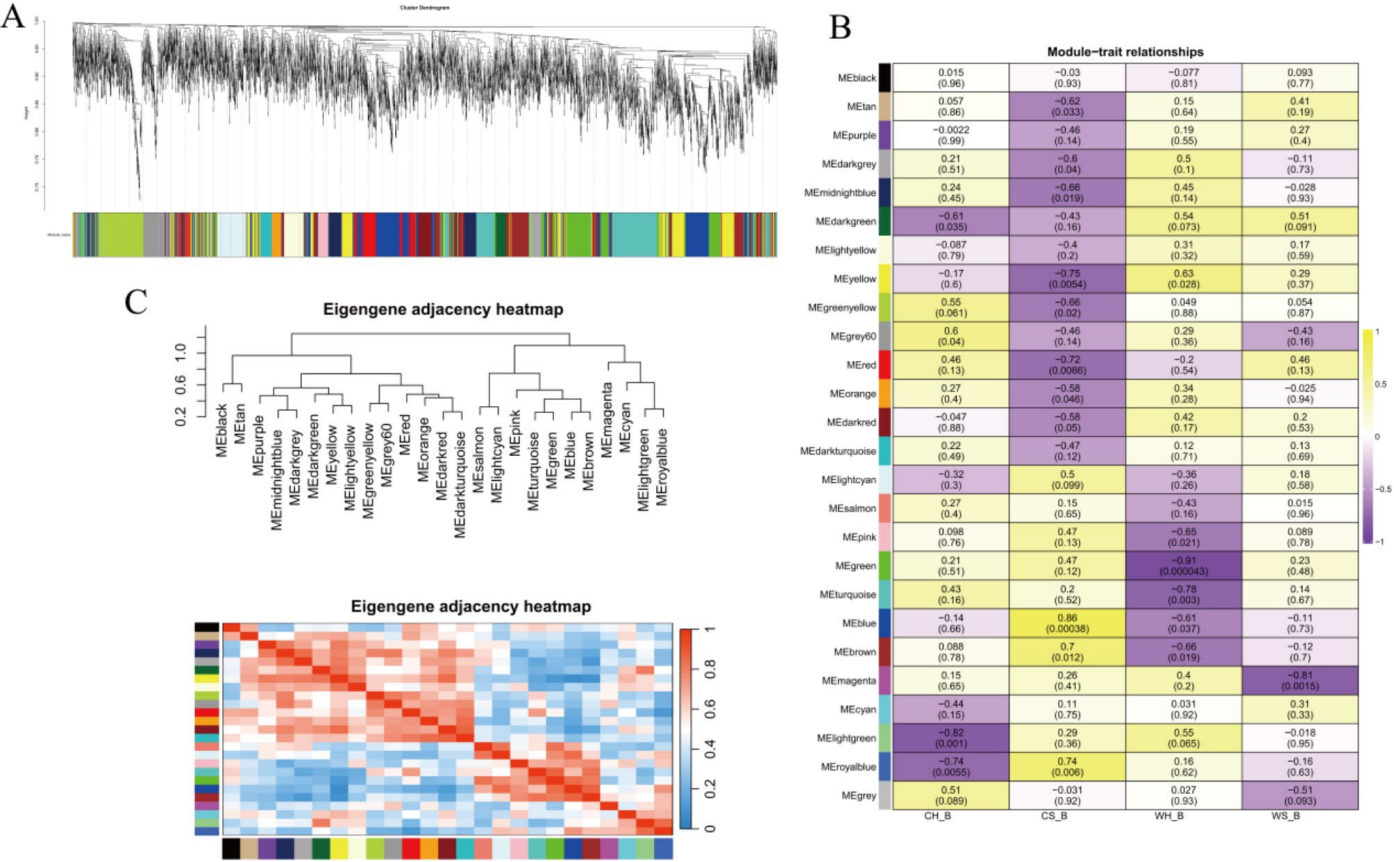
The network construction of weighted correlation network analysis and functional enrichment analysis in BAT. Clustering dendrogram of genes in BAT, with dissimilarity based on the topological overlap and assigned module colors (**A**). Modules associated with 4 groups in BAT. The groups include CH-B (cold and huddling BAT), CS-B (cold and separated BAT), WH-B (warm and huddling BAT), WS-B (warm and separated BAT) (**B**). Eigengene adjacency heatmap shows the correlation between modules. The horizontal and vertical axes represent different modules, with weaker correlations appearing in blue and stronger correlations appearing in red (**C**)

WGCNA is employed to identify pivotal modules, construct gene co-expression networks and filter hub genes. Combined transcriptome data of BAT, WGCNA was conducted by R software. For BAT, a total of 26 distinct modules were identified. The groups most affected by temperature and huddling were cold separated BAT group (CS-B, highest NST) and warm huddling BAT group (WH-B, lowest NST). Among them, the green module was the most significantly negatively correlated with WH-B (correlation coefficient was − 0.91), and the blue, brown, royalblue modules were significantly positively correlated with CS-B (correlation coefficient were respectively: 0.86, 0.7, 0.74) group (Fig. 5).

**Fig. 6:**
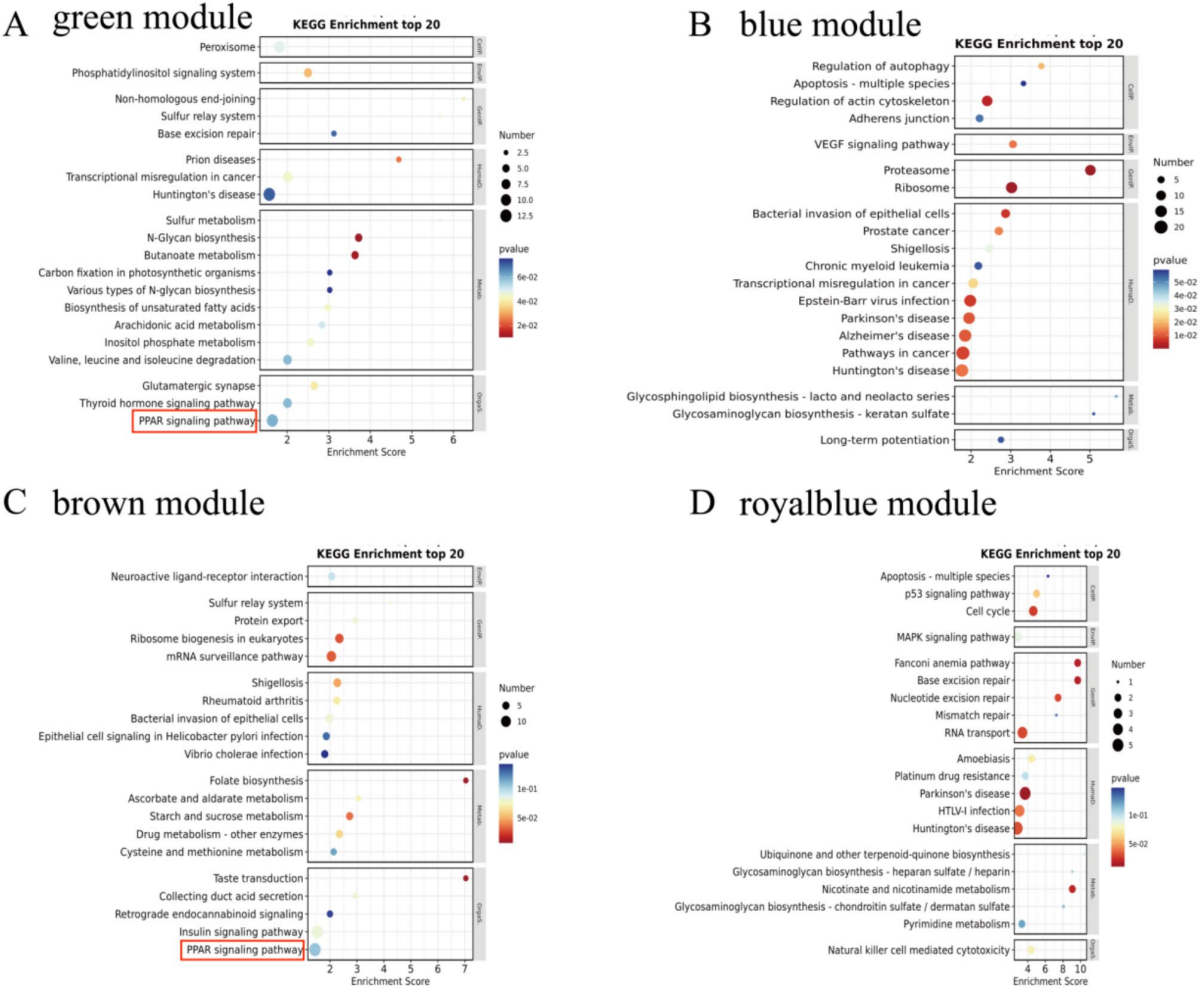
KEGG pathways enrichment analysis of hub genes in different colour modules. KEGG enrichment analysis of genes in the green (**A**), blue (**B**), brown (**C**) and royalblue (**D**) module

For the DEGs contained in the 4 colour modules (green, blue, brown and royalblue) that were significantly correlated with groups, we conducted a KEGG enrichment analysis in BAT (Fig. 6). The green module was significantly negatively correlated with WH-B, hub genes enriched in Thyroid hormone signaling pathway, Biosynthesis of unsaturated fatty acids, PPAR signaling pathway and so on, which are strongly associated with thermogenesis. The blue, brown and royalblue modules were significantly positively correlated with CS-B, hub genes enriched in MAPK signaling pathway, Insulin signaling pathway, PPAR signaling pathway. The results also demonstrated PPAR signaling pathway is a key thermogenic pathway.

### mRNA expression of vital genes in calcium signaling pathway

**Fig. 7:**
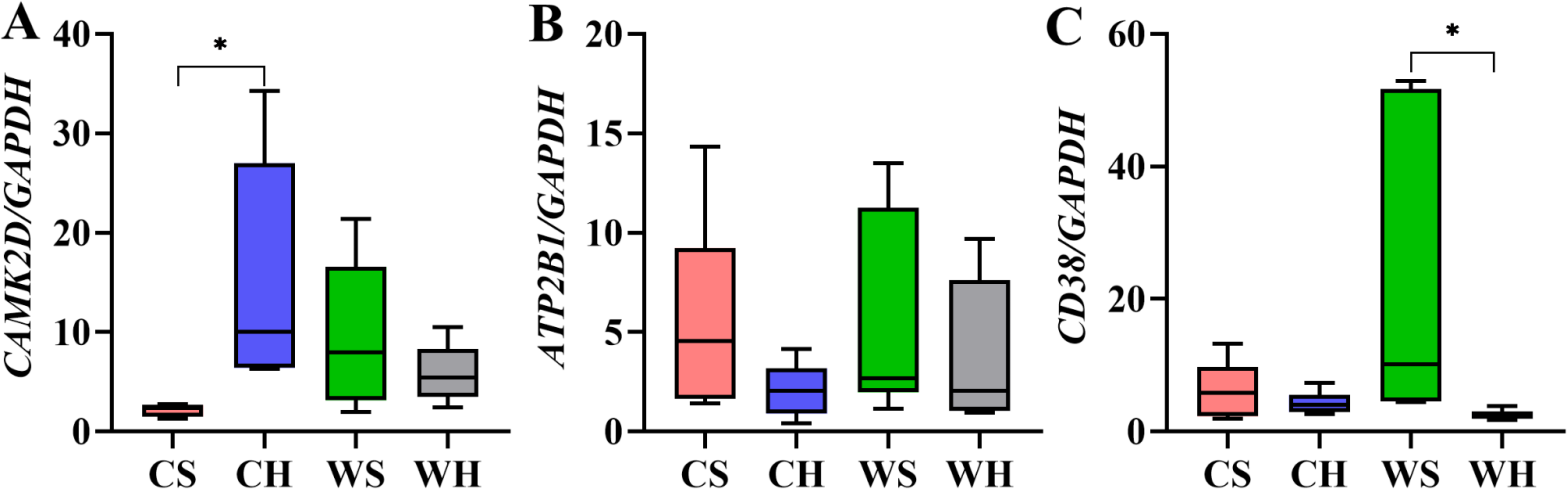
The mRNA expression of key genes of Calcium signaling pathway in hypothalamus. The mRNA expression of (**A**) calcium/calmodulin dependent protein kinase II delta (CAMK2D), (**B**) plasma membrane calcium-transporting ATPase 1 (ATP2B1), (**C**) cyclic ADP-ribose hydrolase 1 (CD38) of Calcium signaling pathway in hypothalamus

Based on the KEGG enrichment pathway results in the hypothalamus, we have chosen three vital genes within the Calcium signalling pathway to evaluate mRNA expression. Calcium/calmodulin-dependent protein kinase II delta (CAMK2D) is responsible for numerous second messenger effects of Ca^2+^. In this study, the expression of CAMK2D was not significantly affected by temperature (F_1,20_=0.112, *p* = 0.741) or huddling (F_1,20_= 2.654, *p* = 0.119). However, there was an interaction between the two factors (*p* = 0.008, Fig. 7A). Plasma membrane calcium transporting ATPase 1 (ATP2B1) catalyzes ATP hydrolysis and transports calcium from the cytoplasm to the extracellular space, thereby maintaining intracellular calcium homeostasis. However, the findings show that neither the temperature (F_1,20_=0.190, *p* = 0.668) nor huddling (F_1,20_=2.457, *p* = 0.164) had a significant effect on the expression of this gene. Additionally, there was no interaction between the two factors (Fig. 7B). Cyclic ADP-ribose hydrolase 1 (CD38) is a multifunctional membrane protein capable of regulating numerous biological functions via intracellular calcium ion concentration regulation. The expression of *CD38* was not significantly affected by the temperature (F_1,20_=2.088, *p* = 0.214). However, huddling can lead to a significant decrease in gene expression (F_1,20_=4.941, *p* = 0.038), and there was no interaction between the two factors (Fig. 7C). In conclusion, among the indicators we selected, temperature alone had no significant effect on the genes of Calcium signaling pathway, but huddling could affect the expression of *CD38*.

### Heat map of candidate genes related to thermogenesis

**Fig. 8:**
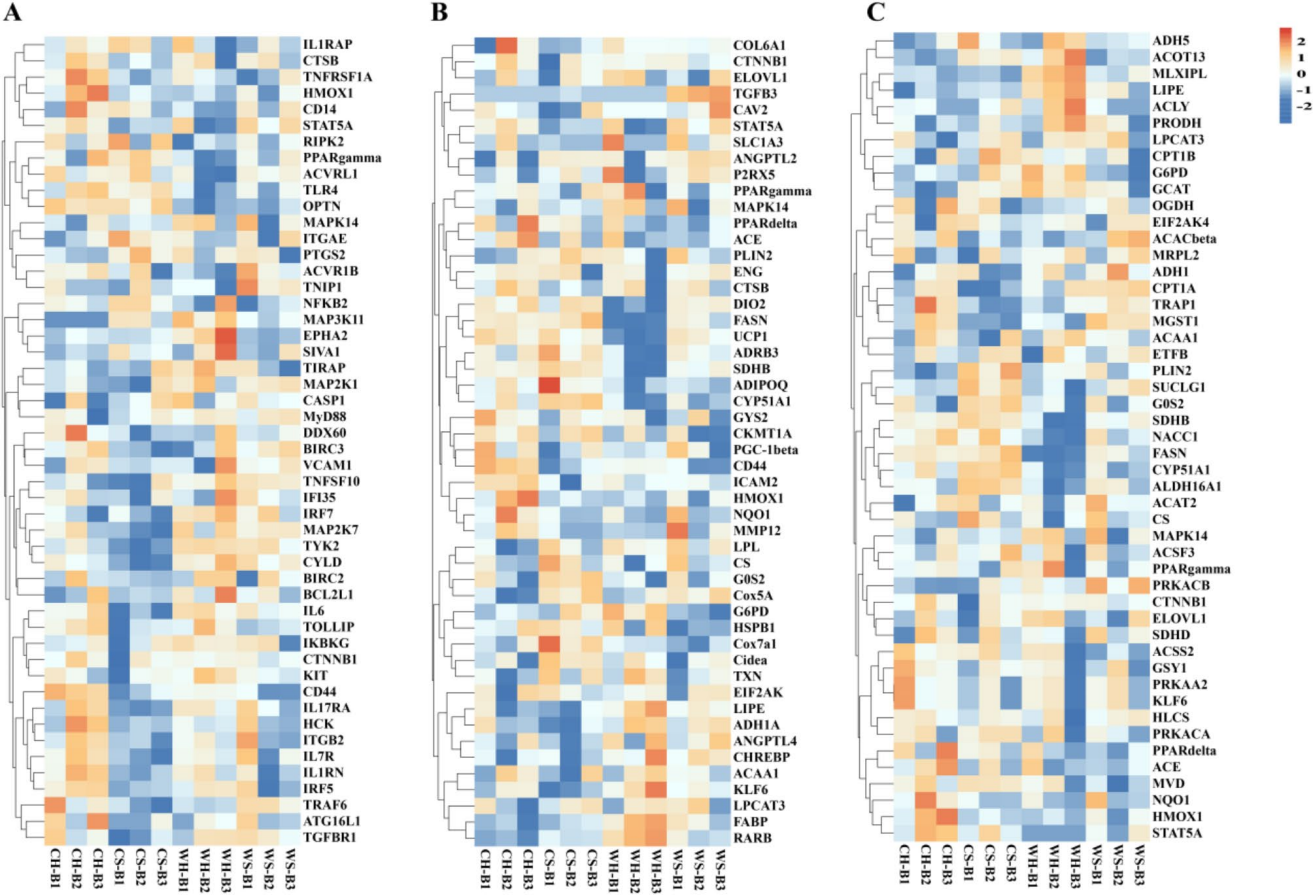
Clustering heat maps of candidate genes related to thermogenesis. Heat map of candidate differentially expressed genes (DEGs) in NF-κB signaling pathway (**A**), Heat map of candidate DEGs in Thermogenesis pathway (**B**), Heat map of candidate DEGs in Fatty acid metabolic pathway (**C**). The red color represents higher expression, the blue color represents lower expression. The cluster analysis of gene expression is based on log2 FPKM (fragments per kilobase of exon model per million mapped fragments) data

As the PPAR signaling pathway could play an crucial role in BAT, we centred our study on this pathway to identify additional pathways that may be related to NST. Ultimately, we discovered three pathways that are associated with NST, NF-κB signaling pathway, Thermogenesis, and Fatty acid metabolism. Essential genes in three pathways are identified and clustering heatmaps based on transcriptome data are plotted using MeV (Multiple Experience Viewer, version 4.9.0) software. We have selected the essential genes (TOP50) from the three pathways as the foundation for further analysis. It is possible that the small number of genes results in an insufficient clustering effect. Based on the clustering results, NF-κB signaling pathway is comprised of DEGs such as *CD44*, *NF-κB2*, *IL6*, *TGFBR1*(Fig. 8A), while the Thermogenesis pathway mainly include *PPARγ*, *UCP1*, *DIO2*, *ADRB3* and *Cidea* (Fig. 8B). Additionally, the DEGs in Fatty acid metabolism pathway mainly involve *ACACβ*, *CPT1A*, *CPT1B*, *FASN* (Fig. 8C).

### The expression of inflammatory factors in BAT, the content of proteins and inflammatory factors in serum

**Fig. 9:**
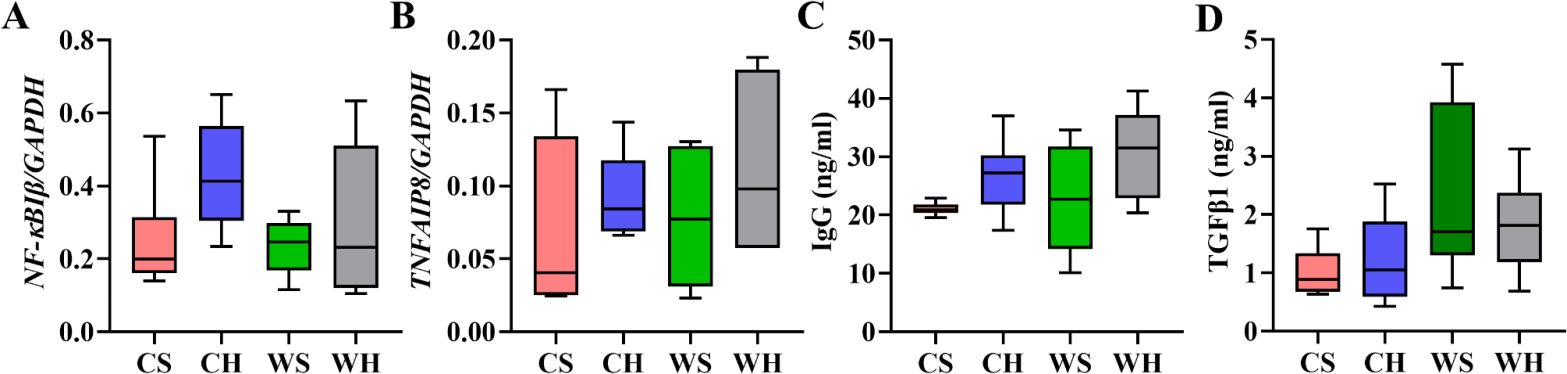
The expression of inflammatory and immune-related indicators. The mRNA expression of nuclear factor kappa-B inhibitor beta (NF-κBIβ, **A**), mRNA expression of tumor necrosis factor, alpha-induced protein 8 (TNFAIP8, **B**), the serum immunoglobulin G (IgG) levels (**C**), the serum transforming growth factor-β1 (TGFβ1) levels (**D**)

NF-κB is instrumental in regulating the immune response to infection, while tumor necrosis factor α-induced protein 8 (TNFAIP8) palys an essential role in regulating inflammation, autoimmunity, and maintaining cellular homeostasis. The expression of *NF-κBIβ*, which is the inhibitor of NF-κB, was not significantly affected by temperature or huddling (Temperature: F_1,20_=1.248, *p* = 0.227; Huddling: F_1,20_=3.766, *p* = 0.067). Similarly, the expression of *TNFAIP8* was not significantly affected by the two factors (Temperature: F_1,20_=0.421, *p* = 0.524; Huddling: F_1,20_=1.806, *p* = 0.194, Fig. 9A-B). Immunoglobulin G (IgG) provides immunoprotection, and temperature had no significant impact on IgG levels (F_1,20_=0.978, *p* = 0.334). However, huddling significantly increased serum IgG levels (F_1,20_=5.482, *p* = 0.03, Fig. 9C). The primary role of transforming growth factor-β1 (TGFβ1) is to monitor the inflammatory process, and exposure to cold significantly reduced serum TGFβ1 levels (F_1,20_=5.966, *p* = 0.024). However, there was no significant effect of huddling on TGFβ1 levels (F_1,20_=0.134, *p* = 0.718, Fig. 9D). In summary, although the two factors did not significantly affect the expression of the two inflammatory genes, huddling behavior exerted specific impacts on serum levels of IgG and cold environment influences the inflammatory factor TGFβ1.

### Expression of UCP1-dependent thermogenesis related genes

**Fig. 10:**
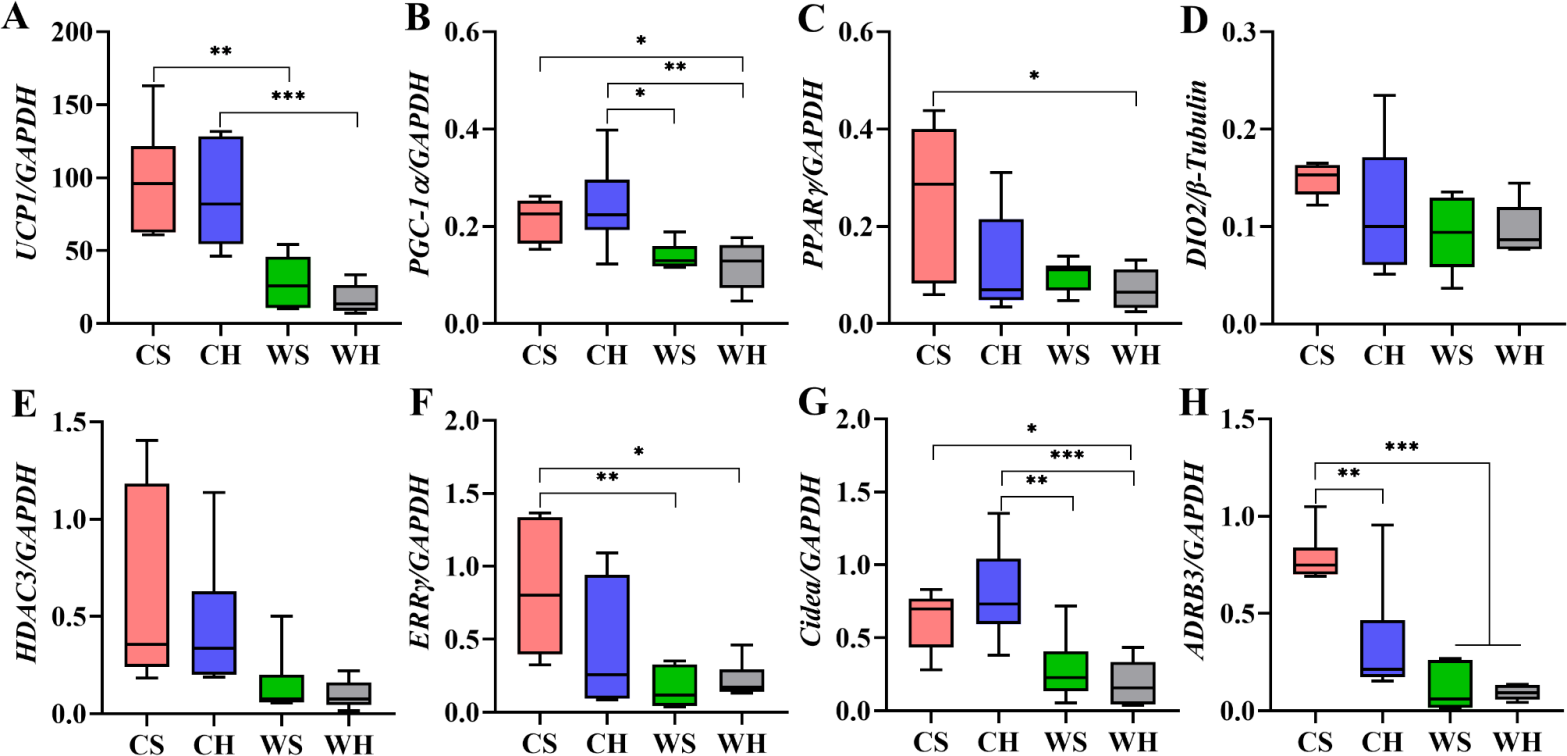
The mRNA expression of thermogenesis-related genes in BAT. The mRNA expression of (**A**) uncoupling protein 1 (*UCP1*), (**B**) peroxisome proliferator-activated receptor gamma coactivator 1-alpha (*PGC-1α*), (**C**) peroxisome proliferator-activated receptor gamma (*PPARγ*), (**D**) deiodinase iodothyronine type II (*DIO2*), (**E**) histone deacetylase 3 (*HDAC3*), (**F**) estrogen-related receptor gamma (*ERRγ*), (**G**) cell death inducing DFFA like effector A (*Cidea*), and (**H**) adrenergic receptor beta 3 (*ADRB3*)

Temperature significantly impacted the expression of the key gene *UCP1* for thermogenesis in BAT (F_1,20_=38.923, *p* < 0.001). Cold significantly increased the mRNA expression of *UCP1*, but huddling did not cause significant changes (F_1,20_=0.921, *p* = 0.349, Fig. 10A). *PGC-1α* (F_1,20_=17.450, *p* < 0.001) and *PPARγ* (F_1,20_=6.331, *p* = 0.021), which regulate *UCP1* expression, were also significantly affected by temperature effect. Cold resulted in increases of mRNA expression of the two genes. Similar to *UCP1*, huddling had no significant impact on mRNA expression of *PGC-1α* (F_1,20_=0.029, *p* = 0.866) or *PPARγ* (F_1,20_=4.047, *p* = 0.058) (Fig. 10B-C). DIO2 is an important regulator of metabolic thermogenesis through thyroid hormones. In this study, it was found that the expression of *DIO2* was significantly affected by temperature (F_1,20_=4.683, *p* = 0.043), while huddling did not have a significant impact (F_1,20_=0.598, *p* = 0.448, Fig. 10D). HDAC3 has the ability to facilitate the activation of BAT, which is vital for maintaining thermogenesis capacity. Cold significantly increased the expression of *HDAC3* (F_1,20_=9.339, *p* = 0.006), but huddling had no significant effect on the gene (F_1,20_=0.204, *p* = 0.656, Fig. 10E). ERRγ may be the key gene for preserving the identity of brown fat and enhancing its quick reaction to cold. Therefore, we measured mRNA expression of *ERRγ*. The results show that temperature had a significantly impact on *ERRγ* (F_1,20_=11.799, *p* = 0.003), but huddling did not significantly affect the expression (F_1,20_=1.683, *p* = 0.209, Fig. 10F). Cell death inducing DFFA like effector A (Cidea) is a marker gene for brown fat. Cold significantly increased the expression of *Cidea* (F_1,20_=24.041, *p* < 0.001), while huddling did not have a significant effect (F_1,20_=0.179, *p* = 0.677, Fig. 10G). ADRB3 is a receptor which has the ability to bind with the neurotransmitter norepinephrine, and its primary function involve the regulation of fat decomposition and thermogenesis. Temperature (F_1,20_=39.258, *p* < 0.001) and huddling (F_1,20_=10.168, *p* = 0.005) had significant effects on *ADRB3* (Fig. 10H). In conclusion, the results indicated that exposure to low temperature can enhance the activation of thermogenic genes we selected. Conversely, huddling behavior did not significantly impact the expression of most thermogenic genes, with the exception of *ADRB3*.

### Expression of key genes in fatty acid metabolism in BAT

**Fig. 11:**
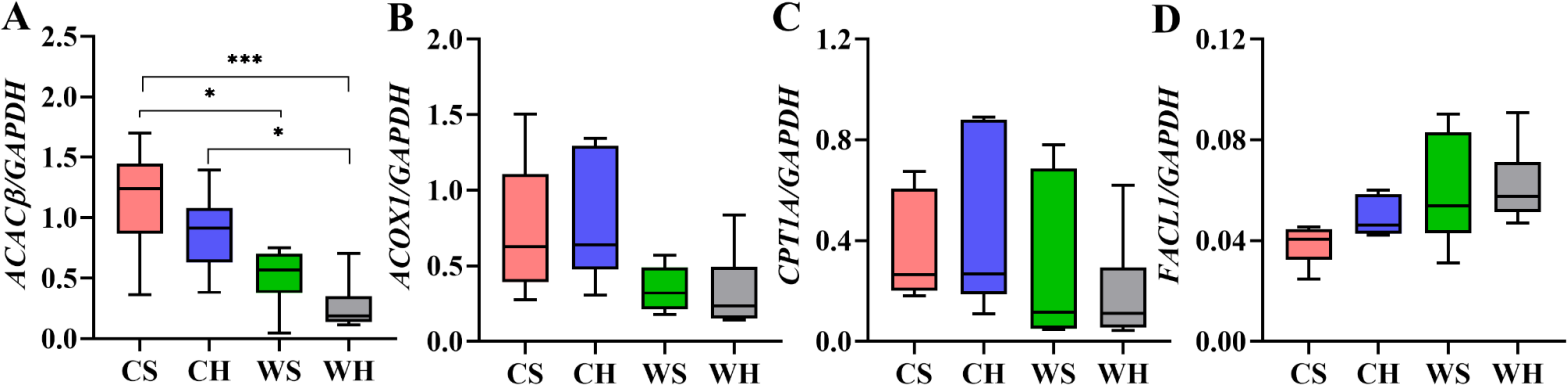
The mRNA expression of Fatty acid metabolism-related genes in BAT. The mRNA expression of (**A**) acetyl coenzyme A carboxylase beta (ACACβ), (**B**) acyl-CoA oxidase 1(ACOXI), (**C**) carnitine palmitoyltransferase 1 A (CPT1A), (**D**) acyl-CoA synthetase long chain family member 1 (FACL1)

Acetyl coenzyme A carboxylase beta (ACACβ) regulates the oxidation of fatty acids. The expression of *ACACβ* was significantly enhanced by cold (F_1,20_=22.266, *p* < 0.001), but huddling had no significant effect (F_1,20_=3.943, *p* = 0.061, Fig. 11A).Acyl CoA oxidase 1 (ACOXI) initiates the process of fatty acid β-oxidation by catalysing the rate-limiting step. The activity of *ACOXI* significantly varied with temperature (F_1,20_=9.405, *p* = 0.006). Nevertheless, huddling had no significantly effect on the expression (F_1,20_=0.015, *p* = 0.903, Fig. 11B). Carnitine palmitoyltransferase 1 A (CPTA1) is a rate-limiting enzyme in the process of oxidizing fatty acids, facilitating the transfer of fatty acids to the mitochondrial matrix for β-oxidation. Temperature (F_1,20_=1.876, *p* = 0.186) and huddling (F_1,20_=0.017, *p* = 0.897) had no significantly impact on *CPTA1* (Fig. 11C). Acyl-CoA synthetase long chain family member (FACL1) catalyzes the formation of fatty acids into fatty acyl CoA, which enters the fatty acid chain β Oxidative pathway. The expression of *FACL1* was found to decrease at the low temperature (F_1,20_=8.009, *p* = 0.01), while huddling did not significantly affect the expression (F_1,20_=1.227, *p* = 0.281, Fig. 11D). In the case of 4 genes we selected, temperature significantly affected expression of the genes in the Fatty acid metabolism except for *CPTA1*, while huddling had no significant effect. These results suggest that Fatty acid metabolism may be more sensitive to temperature changes.

### Expression of associated genes among NF-κB signaling pathway, thermogenesis pathway, and fatty acid metabolism

**Fig. 12:**
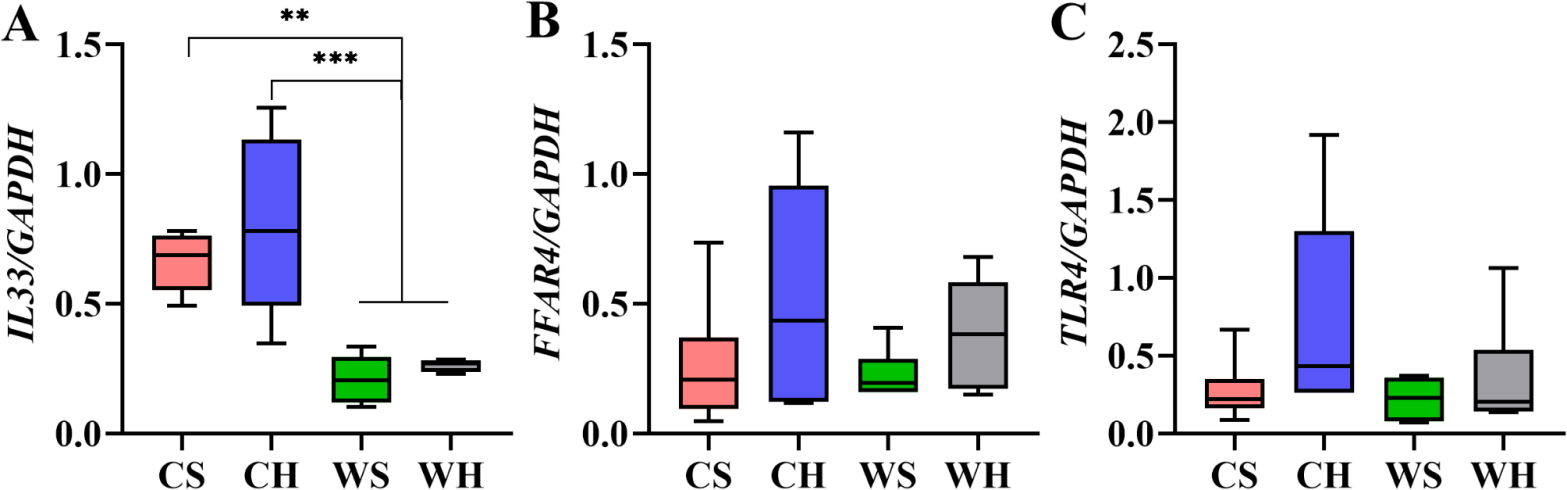
The mRNA expression of genes related to NF-κB signaling pathway, Thermogenesis and Fatty acid metabolism in BAT. The mRNA expression of (**A**) interleukin 33 (*IL33*), (**B**) free fatty acid receptor 4(*FFAR4*)and (**C**) toll like receptor 4 (*TLR4*)

The interleukin 33 (IL33) is the regulator of inflammation associated with both the NF-κB signaling pathway and Thermogenic pathway. Cold significantly increased the expression of *IL33* (F_1,20_=43.605, *p* < 0.001). Huddling had no significant effect on *IL33* (F_1,20_=1.644, *p* = 0.214, Fig. 12A). We had chosen free fatty acid receptor 4 (FFAR4) as the gene linked to Thermogenic and Fatty acid metabolism. FFAR4 is highly involved in adipogenesis, energy metabolism, and inflammation. However, the results of this experiment, indicate that neither temperature (F_1,20_=0.547, *p* = 0.468) nor huddling (F_1,20_=3.436, *p* = 0.079) had a significant impact on the expression of *FFAR4*. Furthermore, no interaction was observed between the two factors (*p* = 0.649, Fig. 12B). We selected Toll like receptor 4 (TLR4) as the candidate gene, as it is associated with NF-κB signaling pathway and Fatty acid metabolism. TLR4 plays a role in the inflammatory response triggered by free fatty acids independent of lipopolysaccharide and its expression is comparable to that of *FFAR4*. The expression of *TLR4* was not significantly influenced by temperature (F_1,20_=1.752, *p* = 0.201) or huddling (F_1,20_=3.333, *p* = 0.083), and there was no interaction between the two factors (*p* = 0.316, Fig. 12C). These results suggest that the association of huddling in several pathways is not significant, but the expression of *IL33* may indicate that inflammation at low temperatures is closely related to thermogenesis. However, the relationship among thermogenesis, fatty acid metabolism and inflammation is poorly correlated. It is necessary to consider indicator selection and more indicators should be selected in the future.

### Protein interaction network analysis

**Fig. 13:**
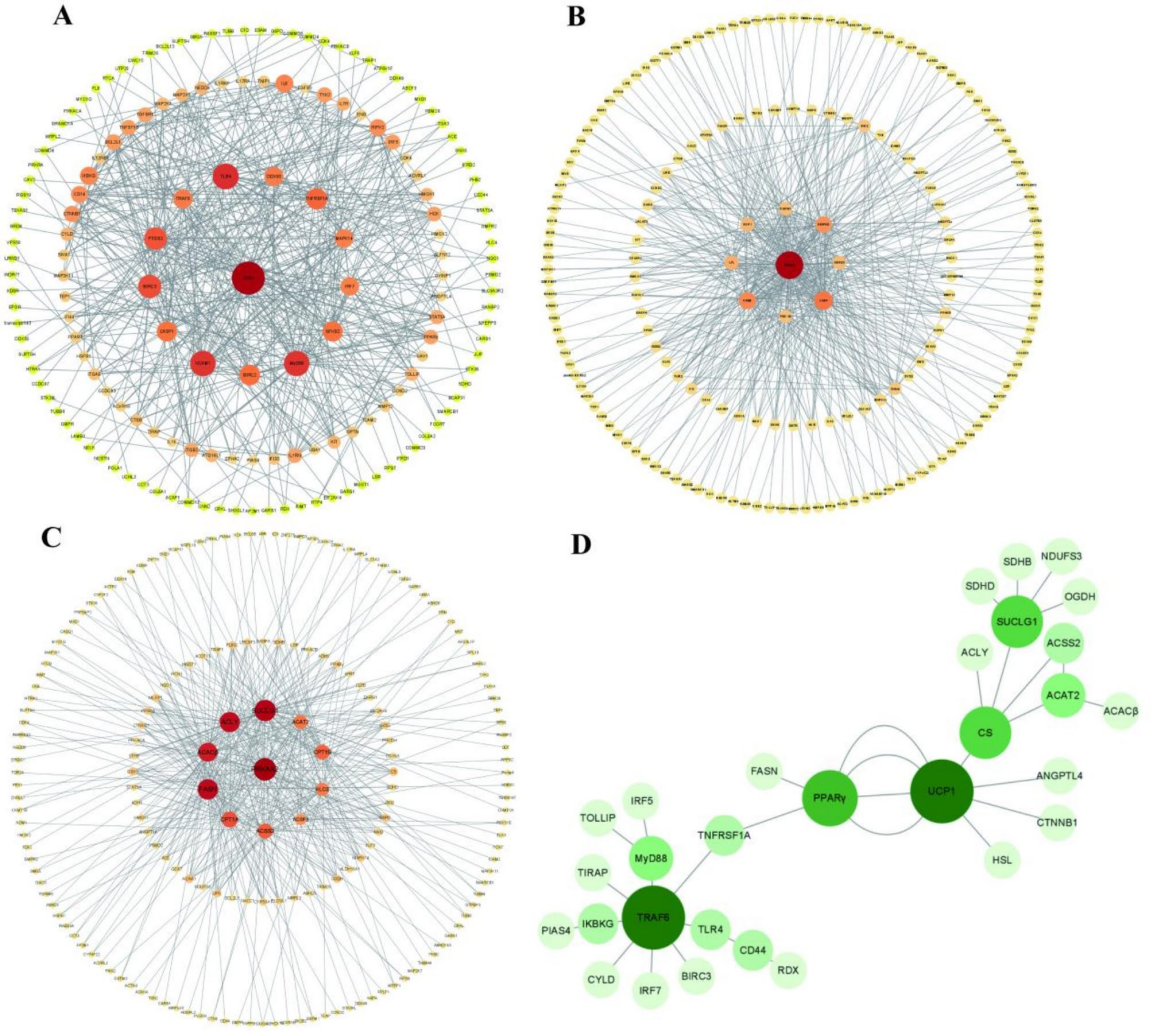
Interactions among differentially expressed proteins (DEPs) in pathways related to thermogenesis. Interactions of DEPs in NF-κB signaling pathway (**A**), interactions of DEPs in Thermogenesis (**B**), interactions of DEPs in Fatty acid metabolism (**C**), interaction of key proteins that connect the three pathways (**D**). Different circles depict various proteins, with lines in the centre indicating potential interactions. The size of the dots increases with the number of associated genes, and the colour darkens

Usually, the implementation of a cell function necessitates the coordinated action of multiple protein molecules rather than the independent action of a single protein. Proteins interact to form a network. This network enables the transmission of biological signals and regulates gene expression and cell life cycles. Therefore, the investigation of protein interactions in this study can improve our understanding about the regulation of thermogenesis, the basic proteins of thermogenesis and the functional links between the proteins in Brandt’s voles. DEGs from the transcriptome data were used to investigate protein interactions within the thermogenesis-related pathways of NF-κB signalling, Thermogenesis and Fatty acid metabolism. Protein-protein interaction (PPI) analyses were performed using Cytoscape software (3.9.0) and the String database. In the NF-κB signaling pathway, CD44 (CD44 antigen) was identified as the central protein in the concentric circle, indicating its high connectivity to other proteins within this pathway. Besides, the proteins in the second circle of the concentric circle were the same. For example, TLR4 (Toll like receptor 4), MyD88 (MyD88 innate immune signal transduction adaptor), in the third circle, for example, IL6 (Interleukin 6), TGFβR1 (Transforming growth factor beta receptor 1) and PPARγ are also key proteins in the NF-κB signaling pathway (Fig. 13A). In the pathway of Thermogenesis, PPARγ could potentially have a significant impact on the overall metabolism and signaling pathways of the system. Key proteins that support PPARγ include UCP1, ADRB3, and PGC-1β. In addition, IL16, TGFβ (Transforming growth factor beta 1), and CD44 work together to coordinate and promote thermogenesis (Fig. 13B). In fatty acid metabolism, PRKAA2/AMPK (Protein Kinase AMP-activated Catalytic Subunit Alpha 2) is a key protein known for its intrinsic energy sensing ability to monitor cell energy levels. Proteins situated in the second concentric circle are significant components of the pathway, including ACACβ, FASN (Fatty acid synthase), CPT1 (Carnitine palmitoyltransferase 1), and CPT1B (Carnitine palmitoyltransferase 1B) (Fig. 13C).

After carrying out the PPI maps of the proteins translated for the DEGs in the three pathways, key proteins for each pathway were identified. However, the execution of a function often involves multiple types of information to be transmitted to each other, which requires connections between pathways. To establish links between pivotal proteins in these pathways, a new PPI map was created by merging genes with high scores in each pathway, in order to demonstrate the interactions between key proteins (Fig. 13D). The darker the green color and the larger the circle in the figure, the more important the protein is. The central pathway connecting the key proteins of the three pathways is roughly TRAF6 (TNF receptor associated factor 6) - PPARγ- UCP1-SUCLG1 (Successe CoA Ligase GDP/ADP Forming Subunit Alpha), TNFRSF1A (TNF receiver superfamily member 1 A), as well as FASN and CS (Citate synthesis), significantly support this pathway as auxiliary effects.

## Discussion

Thermogenesis in rodents has been studied to some extent, but there is a paucity of studies on the relationship between behavior and thermogenesis. Huddling is a cooperative group behavior that allows individuals involved in social thermoregulation to minimize heat loss and thereby reduce energy expenditure, and potentially allow them to reallocate the saved energy to other functions such as growth or reproduction [26]. Previous results from our research group have demonstrated the effect of huddling behavior on NST from a physiological point of view, but there is a lack of in-depth research on the regulation of thermogenesis in Brandt’s voles. The present experiments used Transcriptome sequencing technology to provide new insights into the mechanisms by which behavior regulates thermogenesis in Brandt’s voles. Transcriptome sequencing was performed on hypothalamus and BAT under different temperatures and huddling conditions. All animals maintained a stable rectal temperature during acclimation, indicating they were in normal physiological state. Based on the statistics on the number of DEGs of the transcriptome results, we speculate that BAT was more affected by temperature effects, while the hypothalamus was more affected by huddling. Therefore, we suggest that the low-temperature environment directly influences BAT to produce thermogenesis, while huddling may indirectly affect thermogenesis by impacting the hypothalamus. It is evident that the act of huddling has a potential effect in reducing stress and anxiety caused by cold behavior. Consequently, this may also be a factor contributing to the differential expression of hypothalamic DEGs and it is a psychological effect of huddling. However, psychological effects usually lead to changes in the physiological effects. It has been suggested that the metabolic reduction observed in social huddling animals is influenced by chemical-mediated effects between related individuals in close contact. At ambient temperatures of 10°C, 15°C and 20°C, one study compared the oxygen consumption of mice and Mongolian gerbils grouped in huddled trios, separated trios (animals tested simultaneously but prevented from physical contact) and isolated individuals. Ambient temperatures did not significantly affect oxygen consumption between huddled and separated trios, but were higher in isolated individuals [27]. The authors suggest that significant energy savings can be achieved even without contact, and that other cues from conspecifics (sight, sound, smell, body heat) are also involved in reducing metabolic rate. This may also be one of the reasons why Brandt’s voles have a lower metabolic rate under huddling conditions, as huddling relieves them of stress and anxiety. During acclimation, there was no significant difference in body weight among the four groups, but food intake increased significantly in cold separated group. We suggest that the increase in rest metabolic rate of Brandt’s voles under cold and separated condition is caused by the thermal effect of food. In addition, the difference in capacity of thermogenesis caused by temperature and huddling is an important reason for the change of metabolic rates.

KEGG analysis of DEGs in the hypothalamus indicated significant enrichment of two pathways: Ribosome and the Calcium signaling pathway. Ribosomes play a pivotal role in translating and folding proteins, suggesting that alterations in protein synthesis occur in the hypothalamus in response to variations in temperature and social huddling. Protein synthesis is an energy-intensive process, and in low temperatures, expression of genes associated with protein synthesis declines, which in turn conserves energy. It has been reported that the changes in calcium ions in hypothalamus have the potential to regulate animals’ body temperature. A study of cats found that when Ca^2+^ solution was injected into the posterior region of hypothalamus, leaded to a sharp increase in body temperature [28]. Some studies have measured the concentration of Ca^2+^ in cerebrospinal fluid during fever induced by lipopolysaccharide and IL-1β in rabbits. The findings suggest that intravenous injection of lipopolysaccharide or IL-1β can induce fever reactions in animal. Simultaneously, the concentration of Ca^2+^ in cerebrospinal fluid is significantly increased, indicating that the concentration of Ca^2+^ in cerebrospinal fluid is positively correlated with body temperature. Furthermore, an experiment that reversely demonstrated the correlation between Ca^2+^concentration in cerebrospinal fluid and fever was that aspirin inhibited the fever response, and a significant decrease in cerebrospinal fluid Ca^2+^ concentration was detected [29]. In vitro experiments, there were similar conclusions: IL-1β combined with specific receptors can stimulate the release of Ca^2+^ in the brain [30]. Research in rats demonstrated that the concentration of Ca^2+^ in the dorsal medial hypothalamic nucleus cells was detected under cold exposure conditions. Low temperature led to a decrease in Ca^2+^ concentration [31]. These experimental results together suggest that concentration of Ca^2+^ in central nervous system has an essential function in the process of thermogenesis. The mechanism of the Calcium signalling pathway in the hypothalamus after sensing temperature and huddling effect remains unknown. Three genes with significant differences in the Calcium signaling pathway were selected, and their mRNA expression was assessed. The results showed that the temperature did not significantly influence the expression level. However, huddling had a significant effect on the expression of *CD38*. In terms of selected indicators, huddling has an effect on the hypothalamus. While there is limited research on the influence of huddling on hypothalamic activity, existing studies confirm the significant impact of huddling on this brain region. For example, huddling has been shown to have the potential to enhance hypothalamic neuroregeneration [20].

The results of both conventional DEGs enrichment analysis and WGCNA analysis demonstrated that the PPAR pathway plays a pivotal role in BAT NST, and there are three pathways closely related thermogenic: NF-κB signalling pathway, Thermogenesis and Fatty acid metabolism. NF-κB is a key intracellular nuclear transcription factor involved in the body’s inflammatory response, while physiological inflammation plays a role in increasing thermogenesis. Inflammation-induced thermogenic outcomes depend on pro-inflammatory and anti-inflammatory cytokines being balanced [32]. Although NF-κB and TNFAIP8 have important roles in immune and inflammation responses, the temperature and huddling did not appear to have significant effect on their expression in this study. Consequently, we infer that these two cytokines are not involved in thermogenic regulation or do not rely on mRNA expression changes to influence thermogenesis. The specific cellular and molecular processes which control thermogenesis are yet to be fully comprehended, but current references indicate that there are three molecular mediators responsible for the inflammatory thermogenic response: TLR-4, thermogenic cytokine (IL-1β、IL-6) and thermogenic prostaglandins [33–36]. Studies have found that TLR4 activation and subsequent inflammatory responses are key regulators to suppress adaptive thermogenesis, Obesity-mediated TLR4 activation represses adaptive thermogenesis through endoplasmic reticulum (ER) stress-mediated mitochondrial dysfunction [37]. In addition, deleting TLR4 in POMC neurons is sufficient to increase energy expenditure and thermogenesis in male mice [38]. However, measuring the mRNA expression of *TLR-4* indicated no significant impact from temperature or huddling, implying that this indicator is not a crucial inflammatory factor for physiological inflammatory thermogenesis in Brandt’s voles. Further expansion of the assay is necessary to clarify the inflammatory factors that contribute to thermogenesis in BAT. Measuring serum indicator levels revealed that temperature has an impact on TGFβ1 and huddling affects IgG. Additionally, alterations in immune or inflammatory factors were observed in the serum, indicating the presence of a systemic inflammatory response caused by these two factors. It has been reported that TGFβ1 levels in hypothalamus are crucial in regulating energy homeostasis [39]. Some studies in mice have been found that exogenous treatment with TGFβ1 reduced the thermogenic indicators in the BAT reduced including PGC-1α [40]. Our results show that cold temperature reduces serum TGFβ1 levels, which seems to be consistent with previous research, showing an association between low TGFβ1 levels and increased BAT thermogenesis. We can only speculate about the potential contribution of TGFβ1 to physiological inflammatory thermogenesis as the current results do not allow a direct assessment of the specific involvement of TGFβ1 in the increased NST of BAT. A study investigated the immunomodulatory effects of social isolation in mice, tested immune-inflammatory resilience to bacterial sepsis. They found compared to socially housed mice, housed in social isolation mice showed an increased ability to clear bacterial infection, and provided socially isolated mice with artificial nests to replace their natural huddling behavior reversed the increased resistance to bacterial sepsis [41]. Our results show that huddling induced high IgG levels, and similar to this study is huddling behavior increases immunity. Living separated increases physiological inflammation and thermogenesis. Nevertheless, there are some problems with increased population densities. Indeed, a rise in the population of animals has been demonstrated to result in an increase in pro-inflammatory factors [42], which can cause health risk. Cold exposure can induce systemic changes in lipid metabolism, thus enhancing fatty acid transport in brown adipose tissue through numerous pathways [43]. BAT activation occurs through the binding of norepinephrine to β3 adrenergic receptors located on brown adipose cells. This leads to the stimulation of cyclic adenosine phosphate (cAMP)-dependent signaling cascades. Specifically, this leads to the lipolytic release of non-esterified fatty acids (NEFA) through lipid droplets, mitochondria enriched in BAT, which require fatty acid oxidation to facilitate thermogenesis [44], and an increase in mitochondrial fatty acid oxidation leading to thermogenesis.

In this study, temperature plays an important role in fatty acid β-oxidation indexes except *CPTA1*, while huddling has no significant effect, so we believe that the factor that promotes fatty acid metabolism and causes thermogenesis is temperature. From a metabolic point of view, triglycerides are likely to be the main fuel for oxidation during cold-induced thermogenesis [45]. Studies in rats have shown that cold temperatures increase fatty acid metabolism in skeletal muscle [46]. This is similar to our conclusion, but we did not find a study correlating huddling with lipid metabolism, but insofar as huddling reduces energy metabolism in Brandt’s voles, it is hypothesised that huddling reduces lipolysis, but further validation is needed.

In order to verify the transcriptome results, we selected indicators highly associated with UCP1-dependent thermogenesis for mRNA expression analysis. The expression of thermogenic genes significantly increased in low temperatures, yet the impact on huddling was negligible. These results provide evidence that temperature is the primary driver behind thermogenesis in BAT, and that huddling can conserve energy by regulating protein synthesis in the hypothalamus. However, additional experimentation is necessary to determine the specific type of protein synthesis that is reduced. Additionally, the interaction among NF-κB signaling pathway, thermogenesis, and fatty acid metabolism has been established. To elucidate their correlation, we evaluated the mRNA expression of crucial genes in the connecting pathway. The selected gene linking the NF-κB pathway to Thermogenesis was *IL-33*, revealing a correlation between inflammation at low temperatures and thermogenesis, whereas neither temperature nor huddling affected the gene linking Thermogenesis to Fatty acid metabolism or the gene linking the NF-κB pathway to Fatty acid metabolism, but this is limited to the indicators we choose. We speculate that it is the indicators that result in undetected differences in expression, or perhaps epigenetic influences that cause organisms to express differently, but the genes themselves are not altered, however, in both cases, further expansion of the genes will be necessary subsequently to elucidate the connection among the pathways. To demonstrate the connection among these pathways, we conducted PPI analysis on the proteins translated by the DEGs on the three pathways. But the software can only predict the function. The analysis revealed that the pathways interacted with each other and were linked through TRAF6-PPARγ-UCP1-SUCLG1 to promote thermogenesis in a collaborative effort. The function can be further validated by measuring the expression of protein or gene mRNA on this pathway.

## Conclusion

**Fig. 14:**
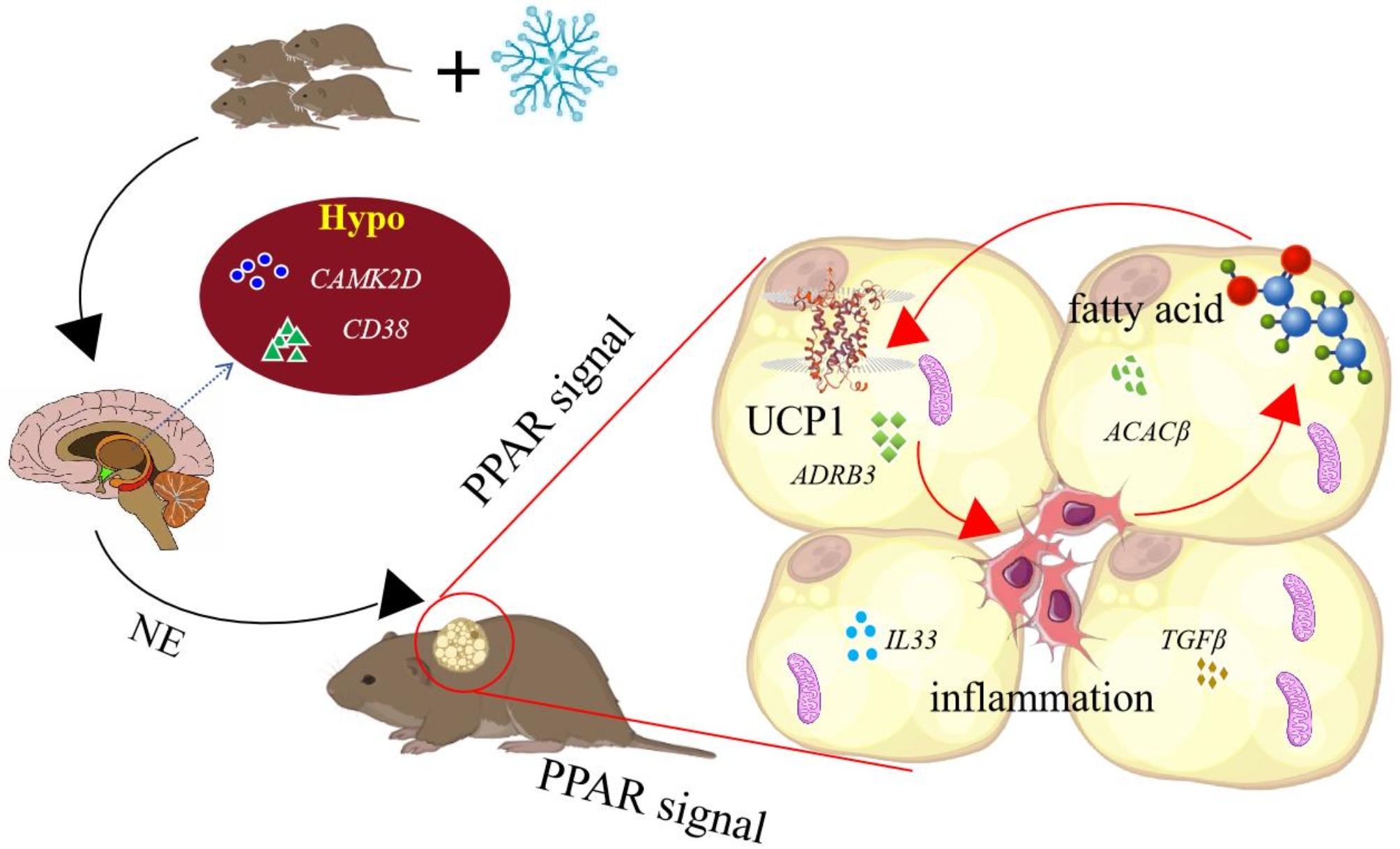
Schematic model of nonshivering thermogenesis induced by cold and huddling in Brandt’s voles

In this study, we explored the BAT thermogenesis with huddling behavior under cold condition in Brandt’s voles. Transcriptome analysis, along with physiological and molecular measurements we selected, reveals that cold and huddling behavior has a substantial effect on BAT thermogenesis. Based on the current results, we hypothesize that huddling has a primary influence on thermogenesis through its effect on Calcium signaling pathway in hypothalamus (Fig. 14).

1. Cold temperature elicited a more pronounced effect on the functional pathway of DEGs enrichment in the BAT, while huddling exerted a more marked effect on the functional pathway of DEGs in hypothalamus. Cold acclimation and huddling had a collaborative influence on the regulation of thermogenic function.
2. Fatty acid metabolism increased during cold acclimation and activated thermogenic pathways. This process was accompanied by an inflammatory response in the cells.
3. Based on transcriptomic data, we predict the hypothalamus and BAT facilitate thermogenesis via the Calcium signaling pathway-PPAR signaling pathway-NF-κB signaling pathways/ Thermogenesis/ Fatty acid metabolism. The specific thermogenic protein pathway comprises TRAF6-PPARγ-UCP1-SUCLG1. And PPARγ-UCP1 has been demonstrated that temperature and huddling are closely related to thermogenesis.

## Materials and methods

### Animals

Brandt’s voles used in the experiments were from laboratory colonies in the Institute of Zoology, Chinese Academy of Sciences (CAS) in Beijing. Adult male voles about 6 months with a body mass of 30–50 g were chose and the voles were housed in plastic cages (42 × 27 × 20 cm^3^) at a light regime of 16 h light:8 h dark (lights on from 4:00 to 20:00) and room temperature of 23 ± 1°C. The cage was divided into 4 equal compartments, which was separated by stainless steel walls with 6 mm holes. The compartments are connected by pathways (7 × 7 cm^2^). For huddling voles, pathways are open so they can move freely. For separated voles, the pathways are closed, but holes in the iron plate allow them to have the possibility of olfactory, auditory and visual contact. There were 80 voles in the whole experiment, we randomly chose 4 animals in one cage, and they had 2 weeks to acclimate to the environment. Individual voles were stained for identification. The voles were fed a standard rabbit pellet chow (containing 18% protein, 3% fat, 12% fiber, and 47% carbohydrate, Beijing KeAo Bioscience Co.) and were provided with water ad libitum.

### Experiment design

**Fig. 15:**
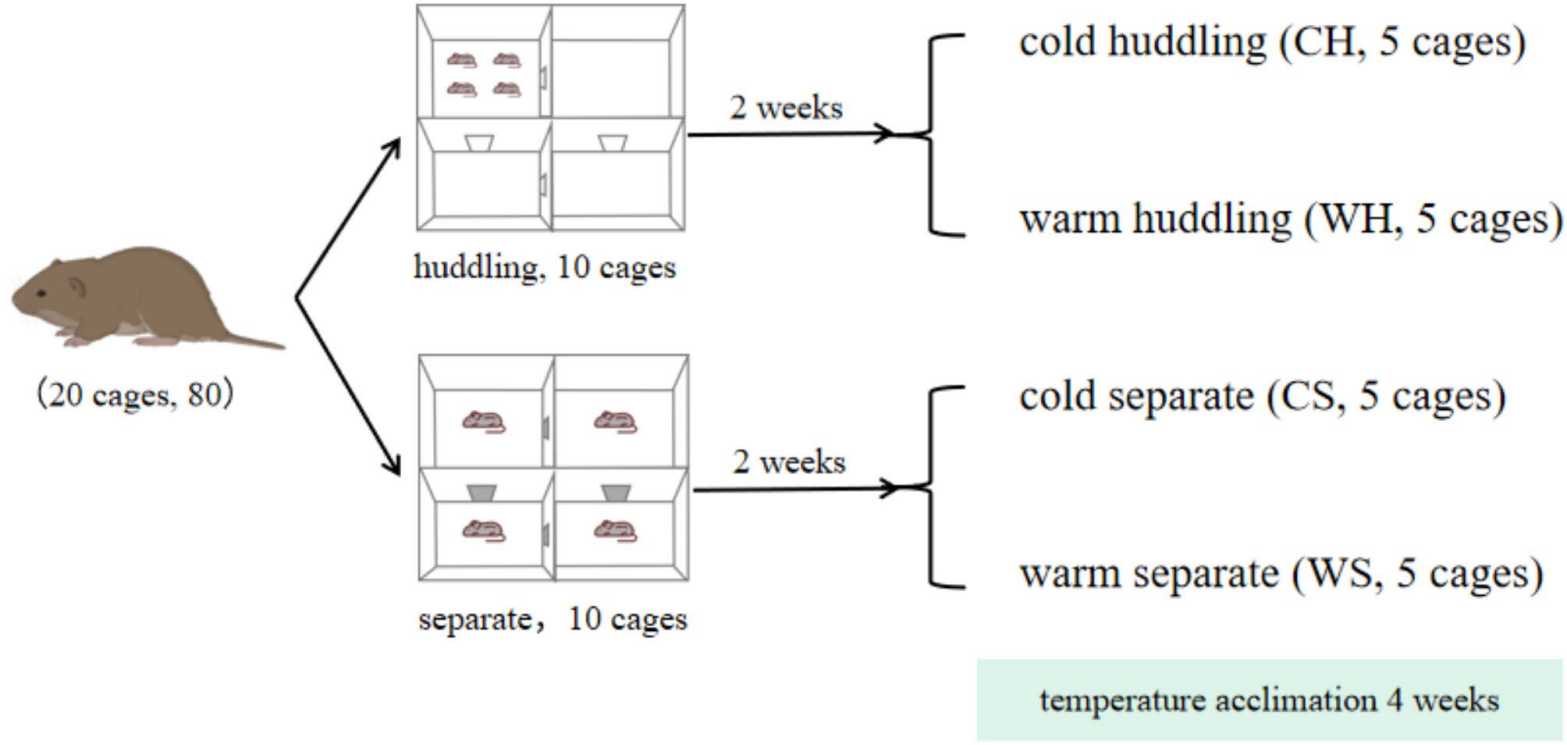
The experimental design

In order to investigate the effects of huddling and temperature on thermogenesis of Brandt’s voles, we set up 4 treatments: cold (at 4 ± 1°C) huddling (CH), cold separate (CS), warm (at 23 ± 1°C) huddling (WH), and warm separate(WS). Each group has 5 cages, and 4 animals are in one cage. All groups were acclimated for 4 weeks (Fig. 15). The animal procedures were approved by the Animal Care and Use Committee of Institute of Zoology, CAS.

### Tissue collection

Fifteen animals from each group were sacrificed, and 3 animals were randomly selected for transcriptome sequencing, 6 animals were randomly selected for qPCR. After the animals were anesthetized with isoflurane, we took blood via eyeballs bleeding, and centrifuged whole blood samples for 30 min at 4 °C with 4000 rpm then froze the serum at − 80 °C. Animals were sacrificed with CO_2_ in the morning. We extracted the hypothalamus and BAT. We peeled the skin with dissecting scissors, opened the skull, and then removed the whole brain. The whole operation was carried out on ice. We carefully removed the hypothalamus from the brain by using ophthalmic scissors. We dissected interscapular BAT for experiments, white adipose tissue was removed from around BAT to avoid affecting the experimental results.

### RNA isolation, library construction, and sequencing

Total RNA was isolated from the BAT and hypothalamus of 12 voles (CH (*n* = 3), CS (*n* = 3), WH (*n* = 3), and WS (*n* = 3)) using TRIzol reagent (R401-01, Vazyme, Nanjing, China). The integrity of the RNA was determined with the Agilent 2100 Bioanalyzer (Agilent Technologies) and agarose gel electrophoresis. The purity and concentration of the RNA were determined with the Nanodrop (Thermo Fisher Scientific) and Qubit (Thermo Fisher Scientific). Only high-quality RNA sample (OD260/280 = 1.8 ~ 2.2, OD260/230 ≥ 2.0, RIN ≥ 8, > 1 µg) was used to construct sequencing library. The clustering of the index-coded samples was performed on a cBot cluster generation system using HiSeq PE Cluster Kit v4-cBot-HS (Illumina) according to the manufacturer’s instructions. After cluster generation, the libraries were sequenced on an Illumina platform by Wuhan Benagen Technology Co., Ltd. and 150 bp paired-end reads were generated.

### Bioinformatics analysis

A total of 4 groups of voles in the experiment, 2 tissues (hypothalamus and BAT) were collected and every tissue had 3 repetitions. We used STAR software (2.7.0d) for sequence alignment, and QoRTs software (1.3.0) for quality control and data processing. We finished quantitative analysis of gene expression by HTSeq software (0.11.2) and analyzed differentially expressed gene by DEseq2 (1.26.0). Genes in hypothalamus and BAT that met the criteria *p* ≤ 0.05, and|fold change (FC)| > 2 were regarded as DEGs. The RNA-seq datasets were released on the Sequence Read Archive at the National Center for Biotechnology Information (BioProject accession number PRJNA1043748). We plotted volcano maps and enrichment pathway maps by Microbiome platform (http://www.bioinformatics.com.cn) and Multiple Experiment Viewer (MeV, 4.9.0) was used to plot clustering heat maps. WGCNA (v1.12.0), implemented in the R program, was used to construct a gene co-expression network for all genes identified in BAT [47]. And used String database combined with Cytoscape (3.9.0) to complete Protein-Protein Interaction Networks (PPI).

### Measurement of serum protein and serum inflammatory factor

We measured concentrations of serum protein and serum inflammatory factor as indicators of inflammation. Serum Immunoglobulin G (IgG) concentrations was determined by ELISA Kit for IgG (CEA544Mu, Cloud-Clone, Wuhan, China) and serum inflammatory factor transforming growth factor-β1 (TGFβ1) was determined by ELISA Kit for TGFβ1 (SEA124Mu, Cloud-Clone, Wuhan, China) were determined, according to the instructions of the supplier.

### Real-time quantitative PCR (RT-qPCR)

We extracted total RNA from the hypothalamus and BAT using TRIzol agent (R401-01, Vazyme, Nanjing, China). After total RNA was isolated, 1 µg total RNA was purified and reverse-transcribed to cDNA using the HiScript^®^ III 1st Strand cDNA Synthesis Kit (+ gDNA wiper) (R312-01/02, Vazyme, Nanjing, China). RT-qPCR analysis was carried out as follows: the cDNA samples (1 µL) were used as a template for the subsequent PCR reaction using gene-specific primers (Supporting information). The final reaction volume of 10 µL contained 5 µL of 2×Taq Pro Universal SYBR qPCR Master mix (Q712-02, Vazyme, Nanjing, China), 1 µL cDNA template, 0.2 µL of forward primer, 0.2 µL reverse primer, and 3.6 µL RNase free ddH_2_O. Each sample was duplicated and the mean value was the expression amount of the sample. RT-qPCR was performed using QuantStudio™ 12 K Flex Software 1.3 (QuantStudio 12 K Flex Real-Time PCR System, Thermo scientific, America). After an initial polymerase activation step at 95°C for 60 s, amplification was followed by 40 cycles (95°C for 15 s and 60°C for 30 s). The reaction was finished by the built-in melting curve. All samples were quantified for relative quantity of gene expression by using GAPDH expression as an internal standard. Relative gene expression was determined by the comparative CT method [48].

### Statistical analysis

We used the software SPSS 26.0 for statistical analyses. Body mass and food intake during the acclimation were analyzed by repeated measures ANOVA or ANCOVA. Differences in RMR between groups were analyzed by two-way ANCOVA with body mass as covariate. The rectal temperature, body surface temperature, mRNA expression, protein levels, serum protein and serum inflammatory factor were analyzed by two-way ANOVA with Tukey’s post hoc tests. The results are presented as means ± SEM, and the level of statistical significance was set at *p* < 0.05. The figures in this article were made by GraphPad Prism 8.0.1.

## Electronic supplementary material

Below is the link to the electronic supplementary material.

Supplementary Material 1

## Data Availability

The RNA-seq datasets were released on the Sequence Read Archive at the National Center for Biotechnology Information (BioProject accession number PRJNA1043748). And the website is https://www.ncbi.nlm.nih.gov/search/all/?term=PRJNA1043748.
