# Supplementary material for: Huddling behavior regulate adaptive thermogenesis in Brandt’s voles (Lasiopodomys brandtii)
Source: Cell Biosci. 2025 Apr 23;15:51. doi: 10.1186/s13578-025-01391-0 (PMC12020165; doi:10.1186/s13578-025-01391-0)
Supplement: Supplementary file 1 — Supplementary Material 1 [file 13578_2025_1391_MOESM1_ESM.doc]

**Verifying transcriptomic results through RT qPCR**

In order to confirm the results of RNA sequencing (RNA-seq), eight DEGs from two tissues were selected for RT-qPCR analysis. We selected proteins involved in regulating thermogenesis and thermogenic proteins in BAT, as well as proteins associated with inflammation and immunity in the hypothalamus for verification. The DEGs from BAT included *UCP1*, *PGC-1α*, *PPARγ*, *ERRγ* (Estrogen-related receptor gamma) and *HDAC3* (Histone deacetylase 3). The DEGs from hypothalamus contains *NF-κBIβ* (Nuclear factor kappa-B inhibitor beta), *PPARγ* and *STAT5A* (Signal transducer and activator of transcription 5A). Although the trends of transcriptome and qPCR results for *UCP1* and *ERRγ* were different in the CS and CH groups, the expression trends of most other genes were consistent with RNA-seq. Therefore, the transcriptome results were considered reliable and could be used for subsequent analysis.


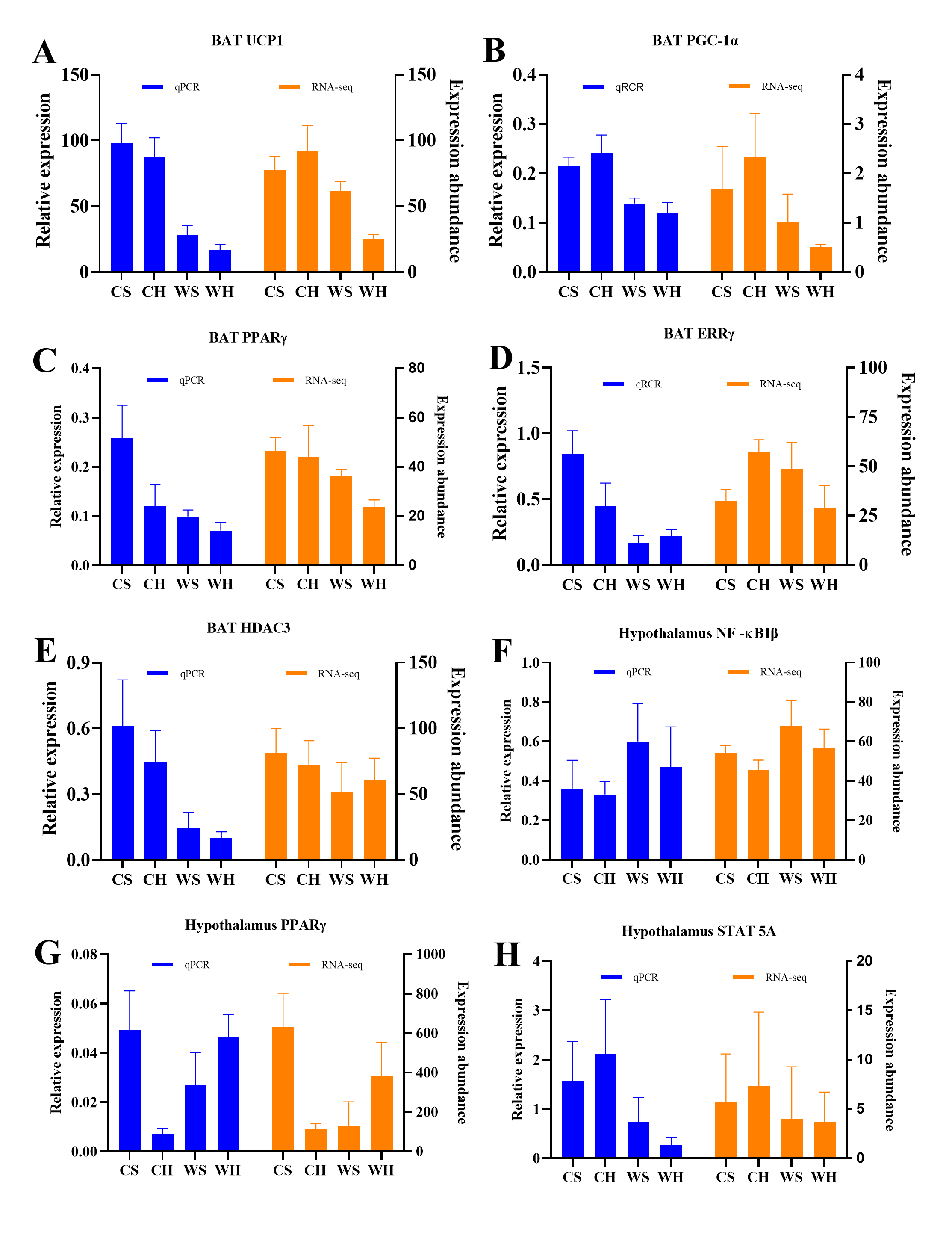


**Figure S1** **Expression abundance and mRNA relative expression of the genes**. The mRNA relative expression and expression abundance of *UCP1* (A), *PGC-1α* (B), *PPARγ* (C), *ERRγ* (D), *HDAC3*(E) in BAT, the mRNA relative expression and expression abundance of *NF-κBIβ* (F), *PPARγ* (G), *STAT5A*(H) in hypothalamus. Blue represents the results of qPCR and orange represents the results of RNA-seq.

**Table S1. The gene-specific primer sequences used for Real-time -qPCR**

| **Primers** | **Forward Primer (5’-3’)** | **Reverse Primer (5’-3’)** |
| --- | --- | --- |
| *NPY* | CGCTCTGCGACACTACATCAA | GGGCTGGATCTCTTGCCAT |
| *AgRP* | GGTGCTAGATCCACAGAACCG | CCAAGCAGGACTCGTGCAG |
| *CART* | CGAGGCCTTTCCCCTAGAGT | CCAGGACTTGCTCCAAGCC |
| *POMC* | TGGATGATGCGTCCCATGA | CGGAATGCGTTTACTCTTGAGC |
| *ERRγ* | CAATCCCCTCCCAACTCCTG | ATAGGTGGCTTTGGGTGTCC |
| *HDAC3* | AAGTTGGAGCAGAGAGTGGC | GTAGAAGTCCACCACCTGGC |
| *NF-κB* | CCTAGGCTCTCTAGGTCCCG | CCATCCTCAGTGACGTAGCC |
| *STAT5A* | TACCTCTTTGGTGCGCAGTC | CTGCAGACTGTCAACTCCGA |
| *CamK 2D* | GGGACCTGAAGCCTGAGAAT | TGCCAGCAAAACCAAACCAC |
| *ATP2B1* | ACCCGAGGAGGAATTAGCAG | ACCACTCGAATCTGTGTTTGGA |
| *CD38* | TGCACAGGTGACAGAAGGTG | GATCAGCCCAGGTTCCAGAC |
| *TNFAIP8* | TGTCCCGCTCTTCTCTCGT | GTGGTGGCGATGGATTTGGA |
| *DIO2* | TGCCTCACTGAAAGACAGGGA | TCAGAAGGGAGGTAACAGGG |
| *Cidea* | AAGTCTCCCAAGCCTAACGC | GGCATCCCACAGCCTCTAAA |
| *ADRB3* | TTGGTAGTGGGACTCCTCGT | AATGCTGGCGGTTACACAGA |
| *ACACβ* | TCAACACAGCCTACGTCACC | GGGTACTTTTCTGGGGAGCC |
| *ACOX I* | AGTGCCAGCATGGTGAAGAA | AATTGAGGCCCACAGGTTCC |
| *CPT1A* | CGTGAGCCGGTACTTGGAAT | GGGTCCAAGATTGACAGCGA |
| *FACL1* | GCCGCGAGTCCTTAAATAGC | CCATGGTTCTGTGCTGGTGA |
| *IL33* | GTCTGCTCCGAGACCTGAAC | AGAACAGGGGAGAAACCACG |
| *FFAR4* | GTACTGAGTGCCGTGGAGAC | GCAGAAGAGGTTGAGCACCA |
| *TLR4* | GTACAGCAGTGGGGAAAGCA | GGAGACAGAGCTGAAAGGGG |
| *UCP1* | ACAGTACCCGAGTGTACCCA | ATGACATTCCAGGACGCCAG |
| *PGC-1α* | ACTCTCAGTAAGGGGCTGGT | ACATGTCCCAAGCCATCCAG |
| *PPARγ* | TGCGCTGGGATTTGAAAGAAG | GTGGGCCAAAATGGCATCTC |
| *GAPDH* | TGCTCCTCCCTGTTTTGGAG | TCCAATACGGCCAAATCCGT |
